# Multiancestry and Multitrait GWAS Meta‐Analysis on Schizophrenia With a Sample of 322,321 Unveils Genetic Links to Chronic Lung Diseases

**DOI:** 10.1111/gbb.70062

**Published:** 2026-07-13

**Authors:** Abudusalamu Ayoufu, Ayinigeer Abulimiti, Wusimanjiang Aierken, ZhouBin Wei, XueCheng Wang, HaiFeng Wang, Liang Zhao

**Affiliations:** ^1^ The Fifth Affiliated Hospital of Xinjiang Medical University Urumqi Xinjiang China; ^2^ Hypertension Center of People's Hospital of Xinjiang Uygur Autonomous Region, Xinjiang Hypertension Institute, NHC Key Laboratory of Hypertension Clinical Research, Key Laboratory of Xinjiang Uygur Autonomous Region “Hypertension Research Laboratory”, Xinjiang Clinical Medical Research Center for Hypertension (Cardio‐Cerebrovascular) Diseases Urumqi Xinjiang China

**Keywords:** chronic pulmonary diseases, genome‐wide association study (GWAS), multiancestry analysis, polygenic risk scoring (PRS), schizophrenia

## Abstract

Schizophrenia (SCZ) is a highly heritable psychiatric disorder, yet its genetic links with chronic pulmonary diseases remain poorly defined. Such links may reflect shared biological pathways and could create opportunities for cross‐disorder risk prediction and therapeutic repurposing. Here we applied a multiancestry, multitrait GWAS framework to SCZ and chronic pulmonary disease datasets. The analysis included 322,321 participants of European and East Asian ancestry from the Psychiatric Genomics Consortium, FinnGen, and 23andMe. We identified 16 previously unreported genetic variants associated with schizophrenia across ancestries. Transcriptome‐wide association analysis and machine learning prioritization highlighted candidate genes, including WBP1L and CNNM2, that may contribute to schizophrenia biology. Gene‐expression‐based drug repurposing further nominated potential therapeutic opportunities shared across psychiatric and pulmonary traits. These findings indicate that schizophrenia and chronic pulmonary diseases share part of their inherited architecture, supporting integrated genetic models for comorbidity, risk stratification, and therapeutic discovery.

## Graphical abstract

This study integrated GWAS data for schizophrenia from European (PGC, FinnGen, iPSYCH) and East Asian cohorts with GWAS of chronic pulmonary diseases (COPD, asthma, ILD, pneumonia) and lung function traits, alongside transcriptomic and proteomic datasets (GEO, GTEx, CommonMind, pQTL). After quality control, we performed meta‐analysis, multitrait GWAS (MTAG), and multiancestry integration (MAMT), followed by fine‐mapping and functional annotation using FUMA, MESuSiE, ANNOVAR, CADD, and RegulomeDB. Gene‐level analyses (MAGMA, mBAT‐combo), TWAS, and SMR identified 38 high‐confidence genes, which were validated via transcriptomic datasets and prioritized using nine machine learning models. Computational drug repurposing (CMap) and protein‐level analyses (BLISS/PWAS) explored therapeutic potential. Multitrait polygenic risk scoring (mtPGS) showed that combining SC with COPD improved predictive accuracy by 8.6%. Overall, we identified 16 novel SC‐associated SNPs and key genes (e.g., WBP1L, CNNM2, IFRD2, PCCB, TRIM8), revealing shared genetic architecture between SC and chronic pulmonary diseases and providing insights for cross‐disease therapeutics and diagnostics.

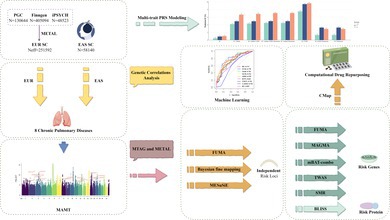

## 1 Introduction

Schizophrenia (SCZ), a psychiatric condition of enigmatic origins and heterogeneous manifestation, is shaped by a tapestry of genetic and environmental elements that influence susceptibility [1]. Its primary manifestations include positive symptoms like delusions, negative symptoms characterized by a lack of drive, and deficits in cognitive function [2]. Annually, SCZ affects about one in every 10,000 adults and is linked to an elevated suicide risk, ranking as the 13th most common cause of suicide globally and curtailing life spans by a decade or two [3, 4, 5].

The etiology of SCZ remains elusive, though it's postulated to be a confluence of genetic, biochemical, and socio‐environmental contributions [6, 7, 8]. Population‐based and meta‐analytic evidence indicates that individuals with SCZ or psychosis have a higher burden of impaired lung function and respiratory diseases [9, 10]. Large primary‐care cohort data further suggest that severe mental illness may precede the accumulation of chronic physical comorbidities over time, including pulmonary conditions [11]. Previous studies have implicated several convergent biological processes across psychiatric and pulmonary disorders, including inflammatory and immune responses, oxidative stress, neurodevelopmental dysregulation, and developmental signaling pathways such as Wnt and Hedgehog signaling [12, 13, 14, 15, 16]. These epidemiological and biological findings indicate a close association between SCZ and chronic pulmonary diseases and raise the possibility that this comorbidity may involve shared genetic mechanisms.

Genetic studies have provided further support for this possibility. Prior genetic correlation, cross‐disorder, and phenome‐wide PRS analyses have shown that SCZ genetic liability is linked to a broad range of nonpsychiatric conditions, including immune‐related and respiratory diseases [17, 18, 19, 20]. However, these studies mainly characterized broad comorbidity patterns rather than specifically dissecting the genetic architecture shared between SCZ and pulmonary disorders. The extent to which shared loci, pleiotropic variants, prioritized genes, biological pathways, and causal relationships contribute to this comorbidity therefore remains unclear [21, 22, 23]. Cross‐trait genetic approaches offer a powerful framework to address this gap by distinguishing shared genetic effects from potential confounding and by identifying biological mechanisms that connect complex diseases [24, 25].

In this pursuit, we probed the genetic nexus between SCZ and chronic pulmonary maladies, leveraging the broadest GWAS datasets from European and East Asian cohorts. Recognizing SCZ's polygenic nature and its biological nexus with respiratory ailments, our meta‐analysis encompassed global SCZ cohorts and indices of chronic pulmonary conditions such as chronic obstructive pulmonary disease, asthma, and pneumonia. Stringent data integrity and fine mapping, with tools like MESuSiE, enabled pinpointing novel variants and loci germane to SCZ. Beyond mere single‐nucleotide polymorphism (SNP) associations, we sought to unearth the underpinning functions and biology. We deployed machine learning algorithms to stratify pivotal genes and harnessed computational drug repurposing for novel therapeutic insights. Ultimately, the mtPGS forged a multitrait PRS model, amalgamating genetic markers across diseases to refine SCZ.

## 2 Methods

Before describing each analysis in detail, we summarized the data sources used across the study. The GWAS meta‐analysis, cross‐trait genetic analyses, fine‐mapping, gene annotation, TWAS, SMR, protein‐based association analyses, and drug‐repurposing analyses were conducted using publicly available GWAS summary statistics, QTL/pQTL datasets, and functional annotation resources. Transcriptome validation and machine learning analyses were performed using independent GEO expression datasets including SCZ patients and healthy controls. The mtPGS analysis was used to evaluate the prediction of SCZ case‐control status in an independent genotype dataset.

### 2.1 Study Participants

Our study incorporates the most expansive set of SCZ GWAS data to date, encompassing patient and control populations from European and East Asian ancestries. For the European cohort, data were derived from 53,386 SC patients and 77,258 controls within the Psychiatric Genomics Consortium (PGC) [26], 6708 patients and 398,386 controls from the FinnGen database (R10 version) [27], and 11,602 patients with 36,921 controls from the iPSYCH GWAS summary available in the GWAS Catalog [28, 29]. For the East Asian analysis, we obtained SCZ GWAS summary statistics from the meta‐analysis conducted by Lam et al. This study combined 20 sample collections from East Asian populations using a two‐stage design, comprising 22,778 SCZ cases and 35,362 controls [30].

Furthermore, our analysis extends to GWAS summary of eight chronic pulmonary diseases and associated biomarkers such as Chronic Obstructive Pulmonary Disease (COPD), Asthma, Interstitial Lung Disease (ILD), Pneumonia, C‐reactive protein, FEV1/FVC ratio, Forced Expiratory Volume, and Vital Capacity, as detailed in Table S1 [31, 32, 33, 34, 35, 36, 37]. All data underwent rigorous quality control checks, including adjustments for genomic control values. Data aligned to the GRCh38 reference were converted to GRCh37 using the liftOver tool for consistency [38].

### 2.2 Meta‐Analysis of GWAS

The meta‐analysis for the European lineage assessed sample overlap and homogeneity of effect sizes through *λ*_meta_ calculations. A *λ*_meta_ substantially above 1 suggested potential heterogeneity between cohorts, whereas a significantly lower *λ*_meta_ hinted at sample overlap. Our analyses maintained *λ*_meta_ values near 1 [39, 40]. For analyses free from sample overlaps, we applied the inverse variance weighted (IVW) fixed‐effects model via METAL software. In cases with sample overlap, heterogeneity indices (*I* ^2^) and *p*‐values from Cochran's *Q* test were evaluated using Metasoft [41, 42, 43], prioritizing random‐effects model outcomes when significant heterogeneity was detected (*I* ^2^ ≥ 50 or P het < 0.05).

### 2.3 Genetic Correlation

Linkage disequilibrium score regression (LDSC) was utilized to independently assess the genetic correlations between SCZ and chronic lung diseases in the European population [44]. Given the smaller sample sizes in East Asian populations, which often yield numerous NaN results in LDSC, we employed GNOVA to compute genetic covariance [45]. These analyses were underpinned by LD scores provided by the 1000 Genomes Project for both European and East Asian lineages [46]. A genetic correlation was considered significant if it met the 0.05 false discovery rate (FDR) threshold.

### 2.4 Smoking‐Conditioned Genetic Correlation Analysis

To assess whether the observed genetic correlations between SCZ and pulmonary phenotypes were attributable to smoking‐related genetic effects, we performed multitrait conditional and joint analysis (mtCOJO) using GCTA [47]. The six pulmonary‐related traits showing significant genetic correlations with SCZ in the European‐ancestry analysis were separately specified as the target traits, and cigarettes smoked per day was specified as the conditioning trait. mtCOJO uses GWAS summary statistics and linkage disequilibrium information to estimate SNP effects on each pulmonary phenotype conditional on the genetically predicted effect of smoking quantity [48]. The resulting smoking‐conditioned GWAS summary statistics therefore represented pulmonary‐trait associations after accounting for the genetic component shared with cigarettes smoked per day. We subsequently applied LDSC to estimate the genetic correlation between SCZ and each smoking‐conditioned pulmonary phenotype, using the same quality‐control procedures as in the primary genetic correlation analysis. The conditional and primary estimates were compared to evaluate whether the genetic overlap between SCZ and pulmonary traits persisted after accounting for smoking‐related genetic effects.

### 2.5 Cross‐Trait Meta‐Analysis

Only pulmonary traits showing significant FDR‐adjusted genome‐wide genetic associations with SCZ were included in the cross‐trait meta‐analysis. MTAG was performed separately for each ancestry using the ‐‐equal_h2 options, which impose equal SNP heritability and perfect genetic correlation across the included traits, respectively [49, 50]. MTAG applies generalized inverse‐variance weighting to combine association evidence across traits while accounting for potential sample overlap through the residual covariance matrix [49, 50]. The ancestry‐specific MTAG results (MT‐EUR and MT‐EAS) for SCZ were subsequently combined using an IVW fixed‐effects meta‐analysis to generate the multiancestry and multitrait (MAMT) results. This constrained MTAG strategy was consistent with that used in a recent large‐scale cross‐trait analysis.

All meta‐analyses explicitly excluded the MHC region (chromosome 6: 25–35 MB) due to its disproportionately large effect sizes [40]. The comprehensive GWAS meta‐analyses calculated *λ*, LDSC intercepts, and effective sample sizes (4/[(1/*N*_case_) + (1/*N*_control_)]) as documented in Table S3.

### 2.6 Fine Mapping

Fine‐mapping analyses were performed to prioritize likely causal variants and to distinguish shared from ancestry‐specific genetic signals. We implemented a significance threshold of 5 × 10^−8^ to delineate independent variants through Functional Mapping and Annotation of Genetic Associations (FUMA) [51]. FUMA facilitated the characterization of genomic risk loci and annotated these variants using LD data derived from the Phase 3 reference cohort of the 1000 Genomes Project tailored for populations of diverse ancestries. We established that the maximum permissible *p*‐value for lead single nucleotide variants (SNVs) should not exceed 5 × 10^−8^, with a stringent cutoff set below 0.05. Independent SNVs were identified with an *r* ^2^ < 0.6, and lead SNVs showed an *r* ^2^ < 0.1 across a 1 Mb span. Genomic risk loci were designated by amalgamating regions where lead SNVs were separated by less than 500 kb [21].

Annotations were performed using ANNOVAR [52], alongside evaluations of combined annotation‐dependent depletion (CADD) and RegulomeDB scores to assess regulatory potential [53, 54]. To validate the reproducibility of these risk loci, a multiancestry statistical fine mapping approach was utilized for all loci demonstrating significant associations within the MAMT analyses [30]. This approach extends traditional Bayesian fine mapping techniques by accounting for heterogeneity across different populations, thereby assigning a lower prior probability to variants exhibiting diverse effect estimates across groups [55]. Each variant's 99% confidence set was ascertained by ordering SNPs within a close LD range (*r* ^2^ > 0.1 of the lead variant) according to their posterior probabilities and sequentially incorporating them until the cumulative posterior probabilities met or surpassed 0.99 [56].

In addition, to investigate whether genomic risk regions are shared across ancestries, we employed MESuSiE for multiancestral fine mapping [57]. Specifically, we established a 500 kb window centered on the most pivotal independent risk loci within each genomic risk region, merging any overlapping areas. Each region was analyzed sequentially, utilizing edge *z*‐scores and SNP–SNP correlation matrices as inputs for fine mapping across all methodologies [57]. We assessed shared and ancestry‐specific signals detected by MESuSiE using a probability of inclusion (PIP) threshold of 0.5 [57].

### 2.7 Gene Annotation

For gene annotation, we utilized FUMA to annotate MAMT summary statistics, extracting both positional mapping and expression quantitative trait loci (eQTL) mapping results from FUMA. The LD reference set employed for gene annotation in FUMA was derived from the 1000 Genomes Project, representing multiple ancestral populations. Additionally, eQTL gene annotations were enhanced using brain tissue datasets available within FUMA [51, 56].

Gene‐based association analyses were conducted using Multimarker Analysis of GenoMic Annotation (MAGMA) and mBAT‐combo [58, 59]. The mBAT‐combo represents a multivariate method that effectively detects gene‐trait associations despite the presence of masking effects, utilizing GWAS summary data to optimize overall efficiency. This approach outperforms the conventional sum‐*χ* ^2^ method, emphasizing its robustness in gene‐trait association discovery [58]. For both MAGMA and mBAT‐combo, significance was determined by a FDR of less than 0.05 after Benjamini–Hochberg (BH) correction. These analyses were used to map associated variants to candidate genes through positional, eQTL, and gene‐based evidence.

### 2.8 Transcriptome‐Wide Association Analysis

Transcriptome‐wide association studies (TWAS) were conducted using FUSION software to identify genes whose genetically predicted expression was associated with SC. The SNP weights employed were sourced from the FUSION website and derived from various external studies. These included SNP weights from all available tissue types such as brain tissues, adrenal glands, pituitary glands, thyroid glands, lungs, and whole blood gathered from the GTEx v8 project. Additional SNP weights from the dorsolateral prefrontal cortex were included, provided by the CommonMind Consortium [60, 61]. For this analysis, a multiancestry LD reference panel was utilized. The significance threshold for transcriptome‐wide associations in this study was set at an FDR of less than 0.05 for BH‐corrected *p*‐values, ensuring rigorous statistical validation of findings.

### 2.9 Gene–Phenotype Correlation Analysis

To elucidate the relationships between gene expression and phenotypic traits, we conducted summary‐data‐based Mendelian randomization (SMR) analyses using MAMT and eQTL. This included utilizing data from diverse sources such as brain tissues from BrainMeta, blood from the eQTLGen consortium, and a comprehensive array of brain tissues from the GTEx project, in addition to adrenal, pituitary, thyroid, and lung tissues, and nine distinct brain cell types from two studies [62, 63, 64, 65, 66]. SMR was employed to test for pleiotropic associations between gene expression levels and complex traits [67]. This method integrates GWAS and QTL data, assessing the impact of gene expression on the traits of interest. A multiple‐ancestry LD reference panel was used for LD estimations. In our analyses, gene–trait associations were deemed significant if the BH‐adjusted FDR was below 0.05 and the HEIDI test result was not significant (*p* > 0.01).

### 2.10 Credible Gene Identification

To establish a robust set of causal genes, we integrated information from various gene mapping techniques. By synthesizing data from FUMA annotation, MAGMA, mBAT‐combo, TWAS, and SMR, we focused on genes that were concurrently identified by all four methodologies, thereby designating them as credible genes.

Further, we conducted phenotypic enrichment analysis to assess the specificity of these credible genes compared to those less substantiated. This analysis was based on the Mammalian Phenotype (MP) ontology from Mouse Genome Informatics (MGI) [68]. By comparing the frequency of genes associated with specific phenotypes within the credible gene set against that in the noncredible set, we could gauge phenotypic enrichment. The Fisher exact test facilitated the identification of significant differences in phenotype associations.

### 2.11 Transcriptome Validation and Machine Learning Analysis

To investigate the expression profiles of target genes in SCZ patients, we utilized mRNA expression data from five datasets on SCZ patients and their healthy counterparts obtained from the Gene Expression Omnibus (GEO) [69], encompassing 196 SCZ patients and 173 healthy individuals (Table S2). We applied the “combat” method from the “sva” package to merge these datasets into a single normalized expression matrix, effectively mitigating batch effects, which we confirmed through box plots and principal component analysis (PCA) [70].

Furthermore, we employed a suite of nine machine learning algorithms—including Support Vector Machine (SVM), Random Forest (RF), K‐Nearest Neighbor (KNN), Gradient Boosting Machine (GBM), LASSO, XGBoost, Neural Networks, Generalized Linear Model (GLM), and Decision Tree—to prioritize the genes based on their diagnostic potential for SCZ. For each algorithm, we calculated the area under the curve (AUC) and generated receiver operating characteristic (ROC) curves to evaluate the predictive accuracy of the gene models.

### 2.12 Computational Drug Repurposing

We extracted TWAS *Z*‐values for the genes identified as credible and utilized these values as proxies for SCZ traits. We employed the connectivity map (CMap) algorithm to discern drugs capable of reversing disease‐specific gene expression profiles. Specifically, we referenced the TWAS association signals of our target genes against the reference maps in the CMap database, which documents changes in gene expression induced by small molecules, providing a basis for drug–gene pair characterization. We restricted our analysis to reference data from the CMap touchstone dataset, comprising around 3000 well‐documented small molecule drugs tested across nine cell lines. We assessed the connectivity between a genome and a drug based on the consistency of the drug in reversing the expression levels of all associated genes. The CMap algorithm quantifies this relationship using the *τ*‐score, where a negative *τ*‐score suggests that the drug normalizes the gene expression linked to the trait. We posit that more negative *τ*‐scores provide robust evidence for the potential repurposing of a drug to treat SCZ [40, 71].

### 2.13 Protein‐Based Association Analysis

To investigate the intricate proteomic landscape in SCZ, we conducted Biomarker Expression Level Imputation using Summary‐level Statistics (BLISS) analysis. This approach diverges from traditional Proteome‐wide Association Studies (PWAS), which typically depend on individual‐level reference proteomes, thereby restricting the use of emerging summary‐level pQTL data available in the public domain [72]. We analyzed European ancestry pQTL using the MT EUR framework with data from deCODE, UK Biobank, and ARIC, and East Asian ancestry pQTL using MT EAS with UK Biobank, applying both Standard PWAS and BLISS methodologies. For proteins showing significant associations across ancestries (*p* < 0.05), we consolidated findings from all sources and conducted a meta‐analysis using the metafor package [73]. Heterogeneity was assessed with the Paule‐Mandel estimator, applying random effects models for *I* ^2^ ≥ 40; otherwise, employing fixed effects models. Proteins with Bonferroni‐corrected *p*‐values below 0.05 were identified as SCZ risk proteins.

### 2.14 Construction of Multitrait PRS Models

Polygenic risk scores were constructed using PGSFusion [74], a web‐based platform integrating 17 single‐trait, multitrait, annotation‐based, and cross‐ancestry PGS methods. European‐ancestry GWAS summary statistics for SCZ, asthma, COPD, and pneumonia were obtained exclusively from FinnGen to avoid sample overlap with the UK Biobank resources incorporated into PGSFusion.

For the multitrait analyses, SCZ was specified as the target trait, whereas asthma, COPD, and pneumonia were separately specified as auxiliary traits, generating three pairwise models: SCZ–asthma, SCZ–COPD, and SCZ–pneumonia. The mtPGS method jointly models the SNP effect sizes of two genetically correlated traits to improve effect‐size estimation for the target trait [75]. Within PGSFusion, the genetic correlation between each trait pair was first estimated using GECKO [76], after which mtPGS estimated the SCZ‐associated SNP weights under a bivariate model using the European LD reference panel. Default parameters were applied, including an LD correlation threshold of *r* ^2^ = 0.2 and a GWAS *p*‐value threshold of 1 × 10^−6^.

For comparison, a single‐trait SCZ PRS was constructed using DBSLMM‐Auto implemented in PGSFusion [77]. DBSLMM‐Auto models SNP effect sizes using a mixture of two normal distributions and estimates their joint effects while accounting for LD. SNP heritability was automatically set to the estimate obtained from LD score regression, thereby avoiding validation‐set‐based hyperparameter tuning. The resulting SNP effect‐size estimates from the three mtPGS models and the SCZ DBSLMM‐Auto model were subsequently used to calculate the corresponding polygenic scores.

## 3 Results

### 3.1 Meta‐Analysis

**FIGURE 1:**
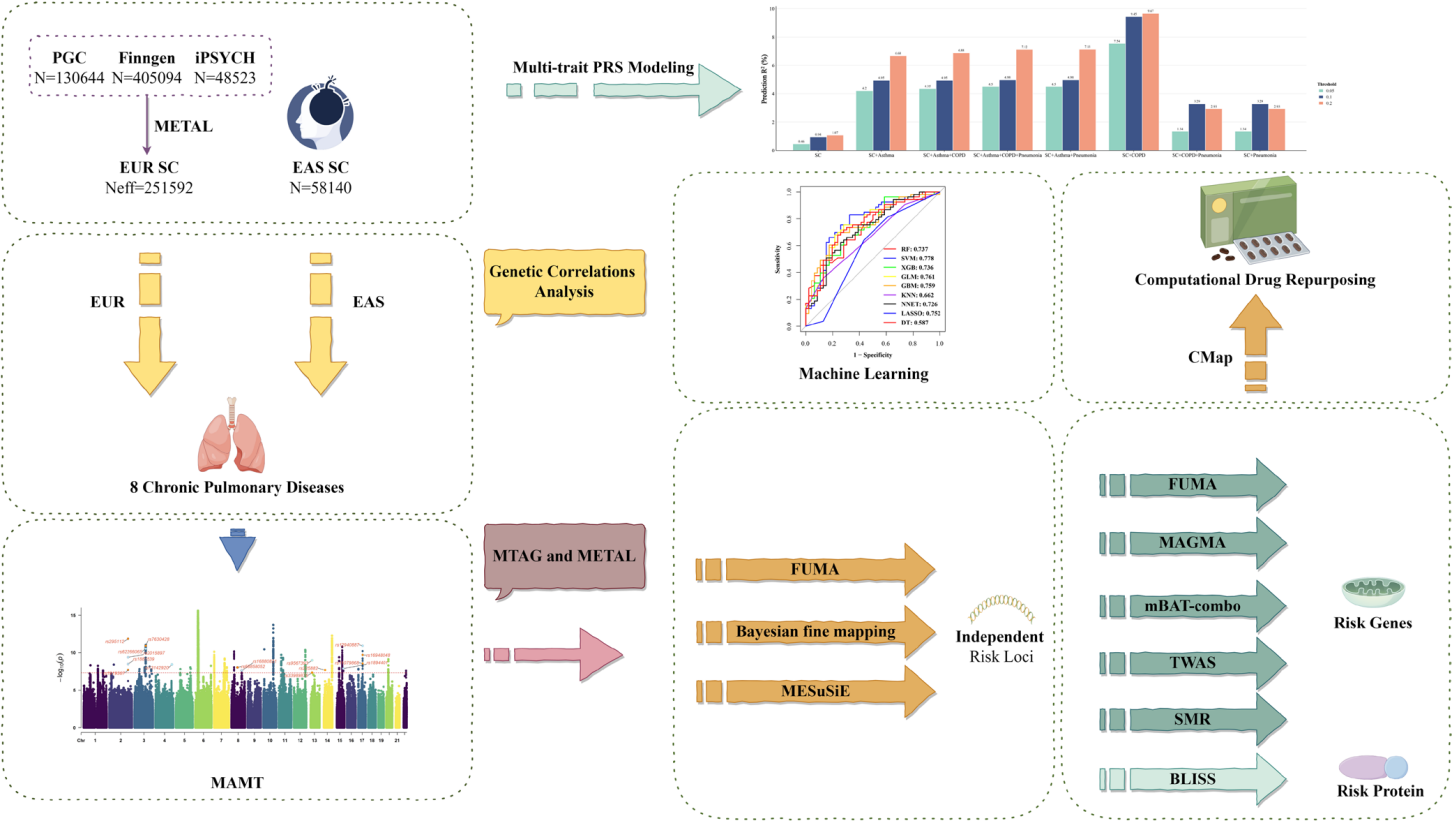
Flowchart. CMap, connectivity map; MAMT, multiple ancestries and traits; MGAMA, multimarker analysis of GenoMic annotation; SC, schizophrenia; SMR, summary data‐based Mendelian randomization; TWAS, transcriptome‐wide association.

The study design and schematic workflow are illustrated in Figure 1. Initially, we computed *λ*_meta_ for each group within the three EUR SCZ GWAS and confirmed no significant sample overlap (*λ*_meta_ > 1) as indicated in Table S3. The meta‐analysis of the MA EUR GWAS data included a substantial cohort with 251,592 valid samples. Notably, for European ancestries other than SC, all displayed significant sample overlap except for two COPD GWAS which indicated a *λ*_meta_ of 0.96.

### 3.2 Genome‐Wide Genetic Correlations

In the European‐ancestry analysis, LDSC identified significant positive genetic correlations of SCZ with asthma (rg = 0.1983, SE = 0.0238, FDR = 2.92 × 10^−16^), COPD (rg = 0.2861, SE = 0.0362, FDR = 6.67 × 10^−15^), C‐reactive protein (rg = 0.0508, SE = 0.0224, FDR = 0.0357), and pneumonia (rg = 0.3849, SE = 0.0398, FDR = 3.41 × 10^−21^). Significant negative genetic correlations were observed for the FEV1/FVC ratio (rg = −0.0480, SE = 0.0217, FDR = 0.0357) and forced expiratory volume (rg = −0.0562, SE = 0.0217, FDR = 0.0192). By contrast, the correlations with interstitial lung disease and vital capacity were not significant (Table S4).

In the East Asian analysis, GNOVA identified significant negative genetic covariance between SCZ and the FEV1/FVC ratio (*ρ* = −0.0156, SE = 0.0047, FDR = 0.0038), forced expiratory volume (*ρ* = −0.0200, SE = 0.0050, FDR = 4.28 × 10^−4^), and vital capacity (*ρ* = −0.0145, SE = 0.0056, FDR = 0.0252). No significant associations were detected for asthma, COPD, C‐reactive protein, interstitial lung disease, or pneumonia. Thus, forced expiratory volume and the FEV1/FVC ratio showed significant negative genetic associations with SCZ in both ancestry groups.

To evaluate the influence of smoking, the six pulmonary traits significantly correlated with SCZ in the European analysis were conditioned on cigarettes smoked per day using mtCOJO. After conditioning, significant positive genetic correlations with SCZ remained for pneumonia (rg = 0.1387, SE = 0.0386, FDR = 8.00 × 10^−4^), COPD (rg = 0.1113, SE = 0.0272, FDR = 2.61 × 10^−4^), asthma (rg = 0.0596, SE = 0.0179, FDR = 0.00135), and C‐reactive protein (rg = 0.0652, SE = 0.0183, FDR = 8.00 × 10^−4^). The negative correlation with the FEV1/FVC ratio also remained significant (rg = −0.0452, SE = 0.0166, FDR = 0.0078). In contrast, the correlation between SCZ and forced expiratory volume was attenuated and no longer significant after conditioning on smoking quantity (rg = −0.0073, SE = 0.0162, FDR = 0.651) (Table S5). These results showed that most of the observed genetic overlap persisted after accounting for smoking‐related genetic effects, although the association with forced expiratory volume did not.

### 3.3 Cross‐Trait Meta‐Analysis

**FIGURE 2:**
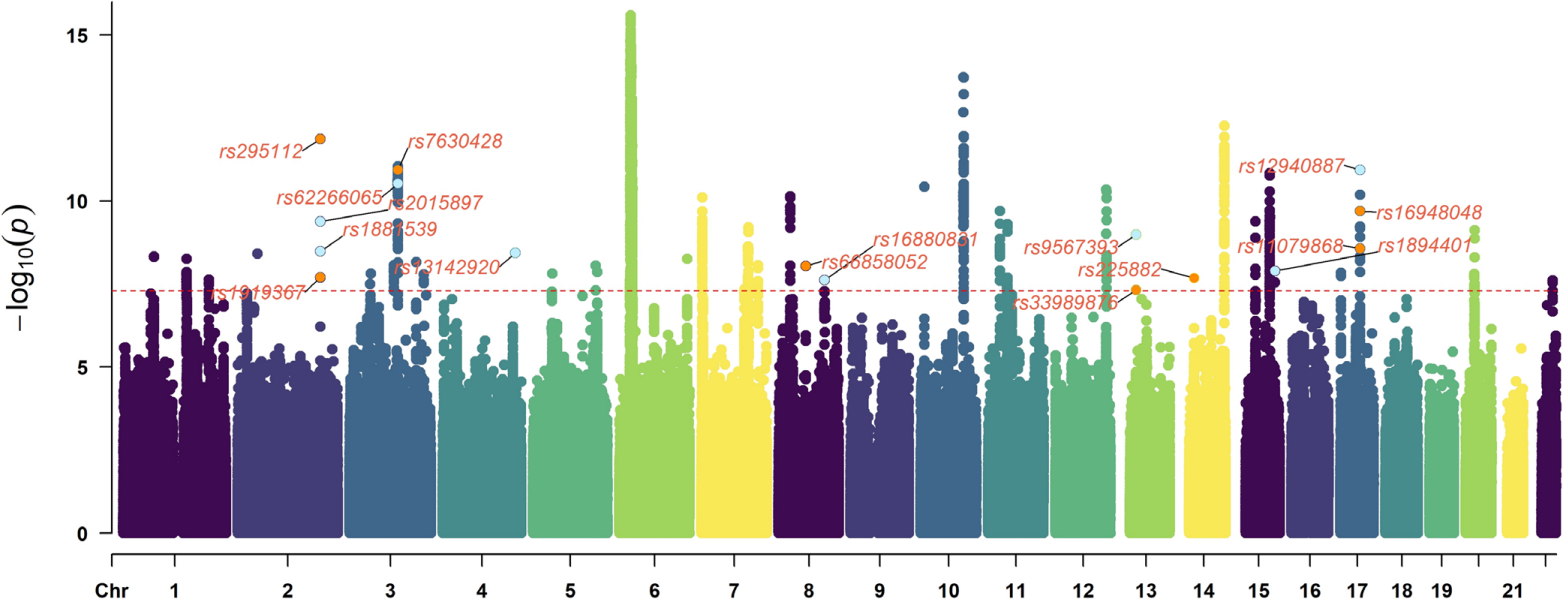
Manhattan plot for MAMT. This figure accentuates the newly discovered single nucleotide variants (SNVs) associated with SC, each situated more than a million base pairs apart from previously reported variants, adhering to a significance threshold of 5 × 10^−8^.

Further, separate multitrait analyses via MTAG assessed MA EUR in conjunction with six chronic lung diseases and traits, as well as MA EAS with three related indicator traits, yielding results for MT EUR and MT EAS, respectively. We then amalgamated these findings using an IVW fixed‐effects model to derive the MAMT results. These comprehensive analyses involved 16,749,693 SNPs and confirmed the absence of residual population stratification (*λ* = 1.048). There was also a robust consistency between the observed *p*‐values and those derived from *Z*‐scores, as shown in Figures 2 and S1.

We identified 159 independent SNPs that reached genome‐wide significance, mapped across 38 distinct loci spaced at least 500 kb apart (Table S6). None of these independent risk loci overlapped with the smoking‐related pleiotropic loci identified by mtCOJO (Table S7). Among these, 16 novel SNVs marked new associations with SCZ, located over 1 million base pairs from previously reported GWAS loci (Figure 2).

### 3.4 Fine Mapping

Utilizing a multiancestry Bayesian fine‐mapping approach, the posterior probabilities for all but nine of these SNVs, including one newly identified variant, exceeded 0.99, indicating a high level of correlation across the genome without signs of multiple independent signals (Table S8).

**FIGURE 3:**
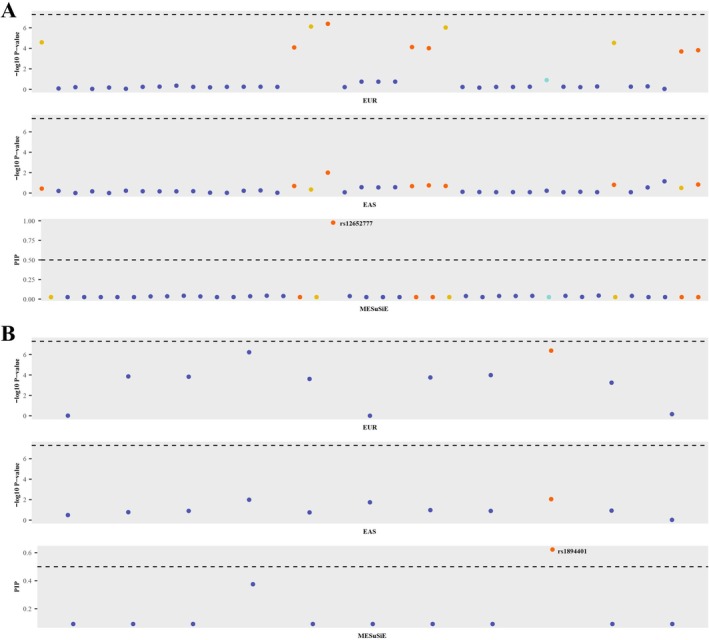
MESuSiE visualization. (A and B) Areas 250 kb above and below rs12652777 and rs1894401, respectively. Blue represents *R* ^2^ of 0, and orange represents *R* ^2^ of 1.

Further analysis with MESuSiE revealed that nine SNPs across seven loci demonstrated signals that were either shared across ancestries or specific to one. Notably, four of these SNPs showed cross‐ancestral shared signals (Table S9). Particularly significant was rs12652777 (PIPshared = 0.88) and rs1894401 (PIPshared = 0.56), both included among the 159 risk SNVs for SCZ, with rs1894401 being among the 16 new discoveries. Rs12652777 exhibited significant correlation with SCZ in the MAMT with a *p*‐value of 1.36 × 10^−8^, though it did not reach significance in the European (MT EUR, *p* = 4.06 × 10^−7^) or East Asian (MT EAS, *p* = 0.01) ancestries independently (Figure 3A). The marginal effect estimates for rs12652777 remained consistent across the two GWAS, suggesting its nonsignificance could be due to its lower minor allele frequency. A similar pattern was observed for rs1894401, an intronic variant within the FES gene, which influences not only lung disease‐related metrics but also has implications for brain volume and other aspects [78, 79, 80, 81, 82].

### 3.5 Genetic Screening

To determine whether incorporating pulmonary traits could improve the identification of SCZ‐associated genes, we performed gene‐level analyses using the cross‐trait‐enhanced SCZ summary statistics generated by the MAMT analysis. Thus, the prioritized genes were identified from SCZ association signals whose statistical estimation incorporated genetic covariance with pulmonary diseases and lung‐function traits, rather than from pulmonary‐disease GWAS alone. Initially, through FUMA annotation, we pinpointed 698 relevant genes (Table S10). Subsequent MAGMA analysis refined this list to 349 targeted genes (Table S11), and further refinement using the mBAT‐combo method identified 202 related genes (Table S12).

**FIGURE 4:**
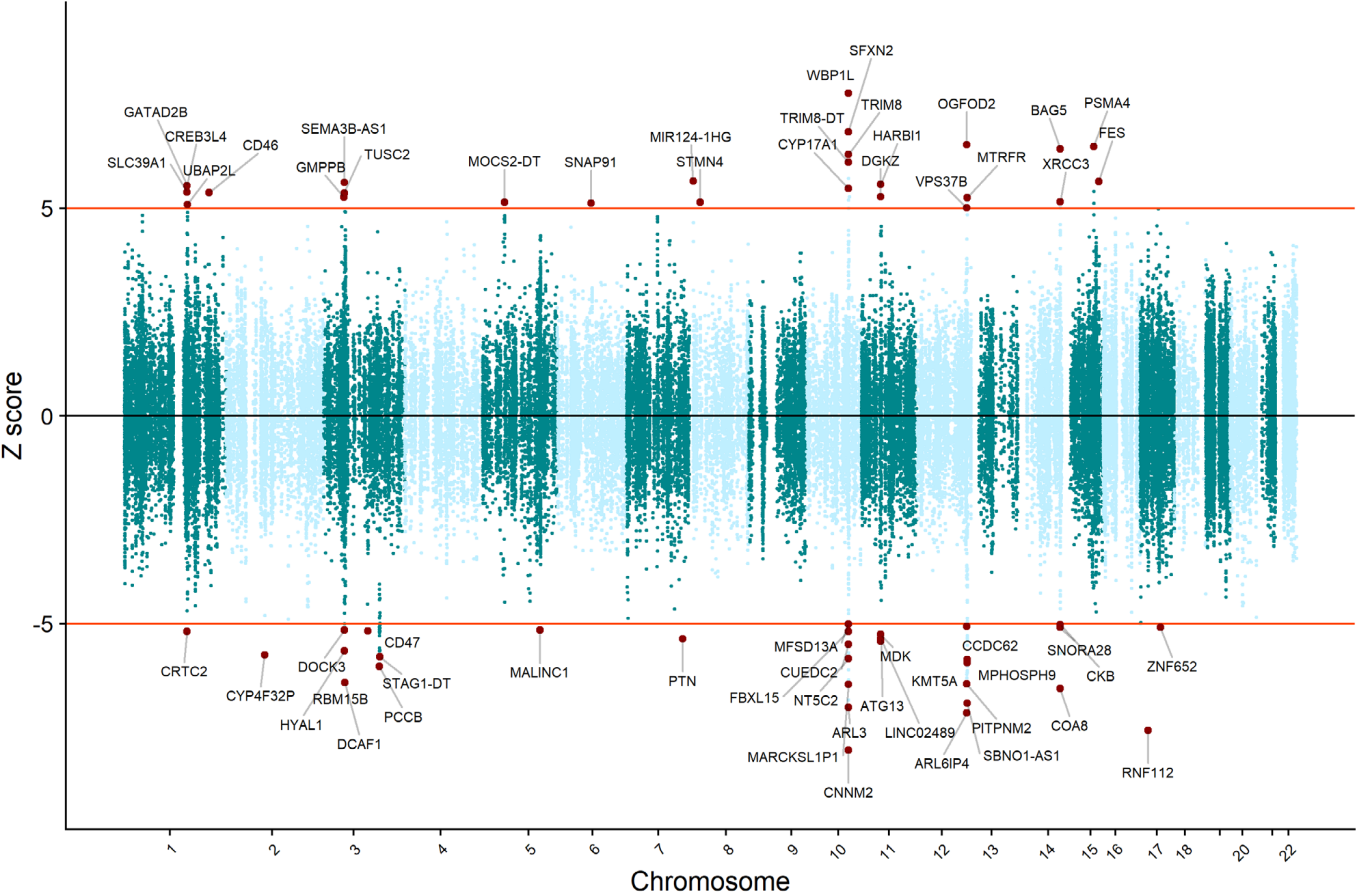
Manhattan plot of *Z*‐scores for genes associated with SC in TWAS significant gene associations are marked with red dots, and the names of genes with (*Z* > |5|) are prominently displayed on both sides of the plot. For genes at the top, increased expression is associated with elevated SC risk, whereas genes at the bottom show a negative correlation with expression.

TWAS highlighted 353 genes significantly correlated with SC (Figure 4 and Table S13), with the most notable being WBP1L (thyroid, *Z* = 7.76, *p* = 8.62 × 10^−15^) and CNNM2 (frontal cortex BA9, *Z* = −8.04, *p* = 8.72 × 10^−16^), corroborating previous findings [83, 84]. Additionally, SMR analysis identified 49 pleiotropic genes, among which GSDMA showed significant negative correlation with SC across multiple tissues, including the brain substantia nigra, adrenal gland, lung, and thyroid (Table S14). MED24 was notably negatively correlated with SCZ in dopaminergic neurons (*β* = −0.05).

**FIGURE 5:**
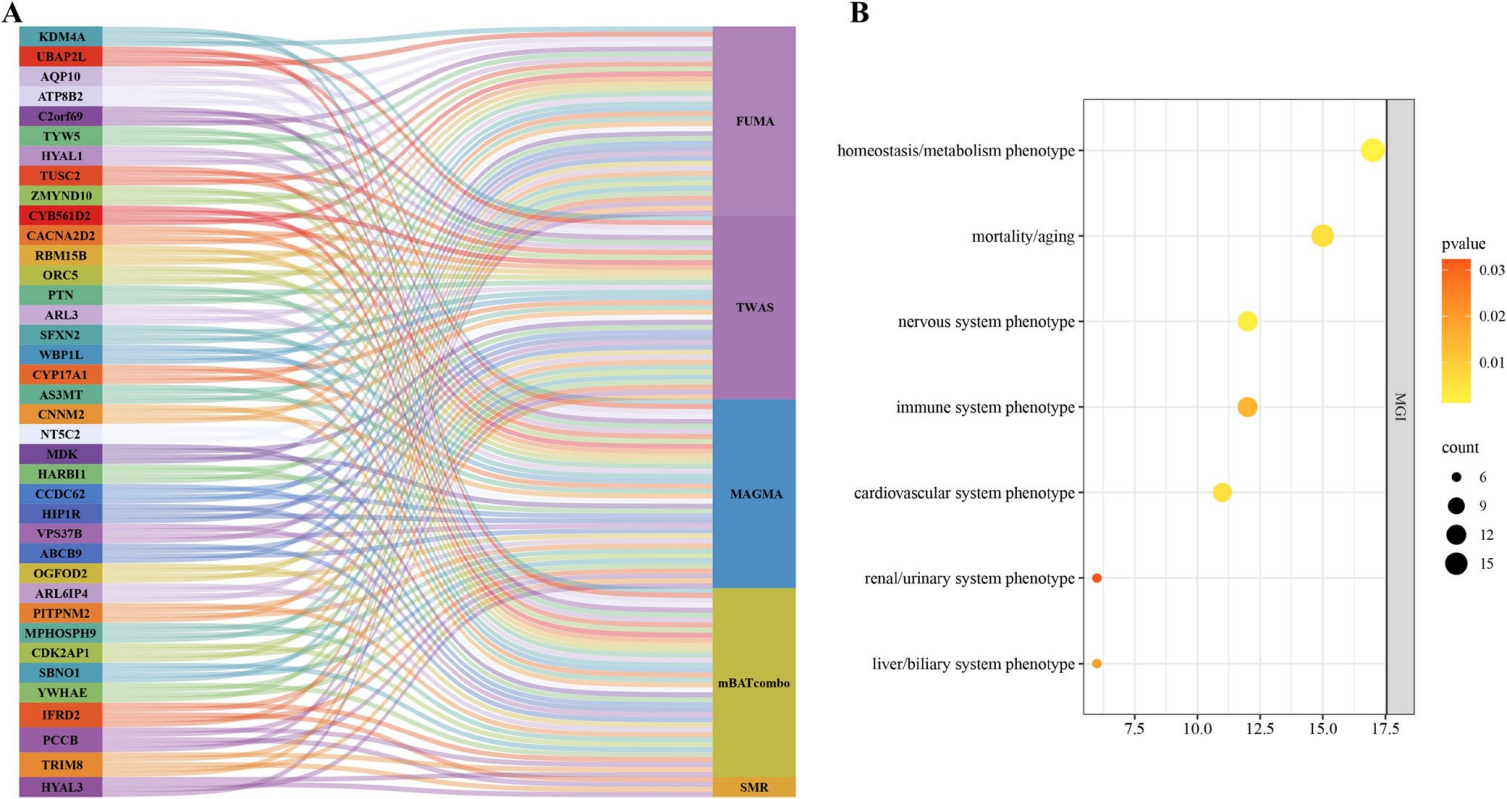
(A) Thirty‐eight SC risk genes identified using five distinct methodologies, each gene recognized by four or more approaches. The left side represents the risk genes, while the right side details the various methods employed. (B) Parallel phenotype analysis of credible SC genes.

Utilizing the aforementioned five analytical methods, we discerned 38 genes recurrently identified in four or more approaches, classifying these as high‐confidence genes. Three genes—IFRD2, PCCB, and TRIM8—emerged in all five methods (Figure 5A). Phenotypic analysis of these high‐confidence genes revealed seven significantly associated phenotypes, highlighting a strong link between metabolic homeostasis and SC, possibly indicative of the metabolic underpinnings of the disorder (Figure 5B and Table S15). Neurological phenotype associations underscore SCZ as primarily a neurological disorder, hinting at potential neurodevelopmental irregularities. Additionally, cardiovascular associations suggest heightened risk of cardiovascular issues in SCZ patients, potentially tied to prevalent lifestyle and stress‐related factors. The linkage with mortality/aging phenotypes underscores an increased general mortality risk, necessitating enhanced preventive and interventional strategies. Associations with immune system phenotypes lend support to theories of immune dysfunction in SCZ, possibly due to inflammation. Connections with hepatobiliary and renal/urinary system phenotypes suggest alterations in metabolic and detoxification pathways, highlighting potential challenges in metabolite clearance [85, 86, 87, 88].

### 3.6 Machine Learning‐Based Gene Prioritization

**FIGURE 6:**
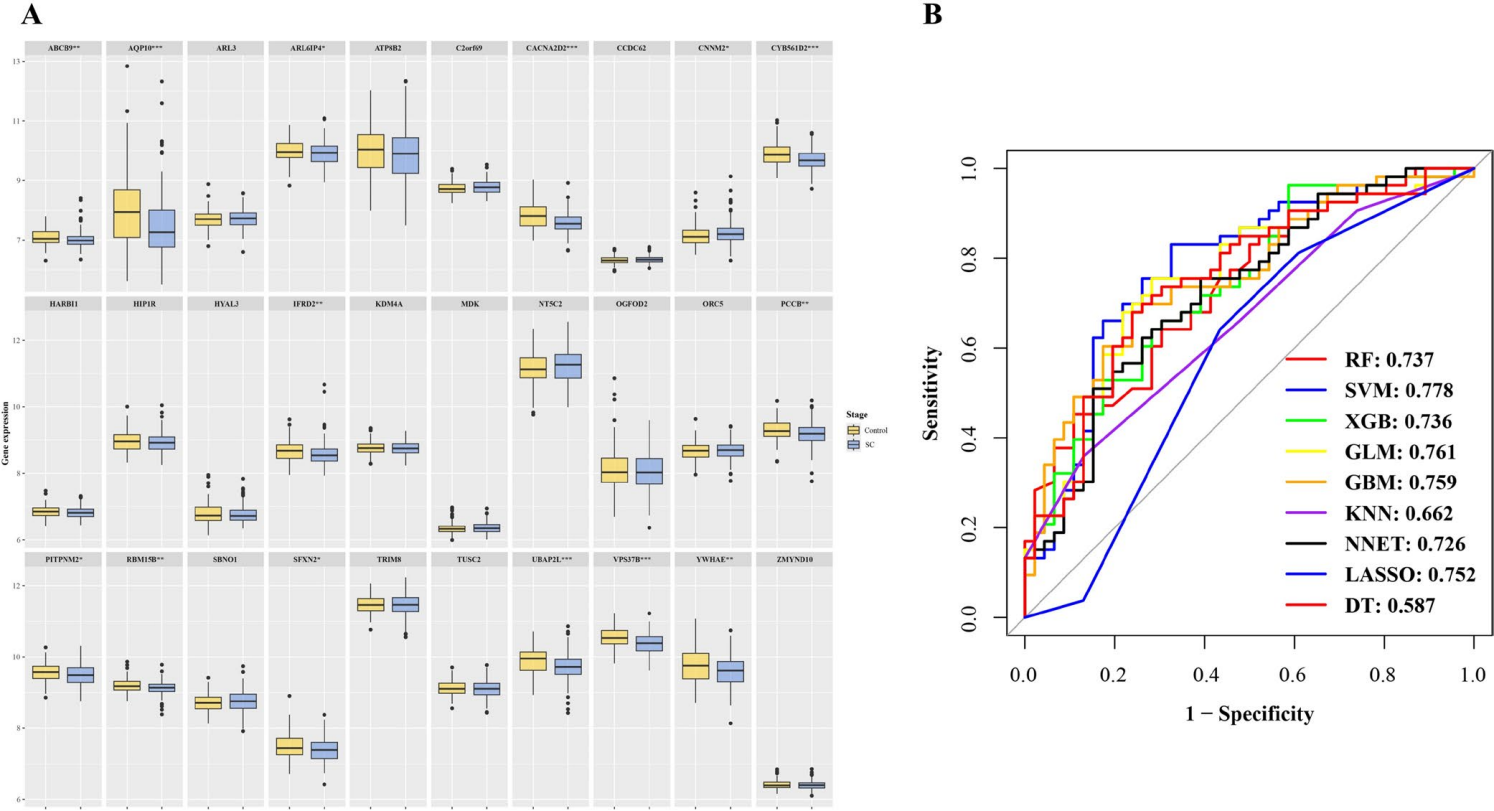
Expression and diagnostic modeling of credible SC genes. (A) Boxplot showing expression differences in transcriptomes for 30 of the 38 credible SC genes. (B) ROC curves for diagnostic models constructed with the 31 credible genes using nine machine learning algorithms. DT, decision tree; GBM, gradient boosting machine; GLM, generalized linear model; KNN, K‐nearest neighbor; NNET, neural networks; RF, random forest; SVM, support vector machine; XGB, XGBoost.

After integrating and correcting for batch effects in five transcriptomic datasets (Figure S2), we analyzed 10,498 genes, including 30 out of the 38 high‐confidence genes identified. A rank‐sum test revealed that 14 high‐confidence genes (46.7%) exhibited significant differences in expression between SCZ patients and controls (*p* < 0.05) (Figure 6A).

Subsequently, we employed nine distinct machine learning algorithms to develop diagnostic models for SCZ (Figure 6B). The SVM model demonstrated the most robust performance, achieving the highest area under the ROC curve (AUC = 0.78). Notably, seven of these models achieved AUC values greater than 0.70, indicating strong diagnostic potential. Particularly, genes such as UBAP2L, AQP10, and ATP8B2 scored highly across several models (Figure S3), underscoring their potential relevance in accurately diagnosing SC.

### 3.7 Computational Drug Repurposing Analysis

We utilized TWAS *Z*‐values from 37 credible genes to conduct a computational drug repurposing (CDR) analysis using the Connectivity Map (CMap) approach, which identified 27 clinically relevant drug classes (Table S16). These classes included adrenergic receptor antagonists and dehydrogenase inhibitors, which corroborate the validity of our results [89, 90]. Additionally, mTOR inhibitors, known to affect neuropsychiatric alterations [91], showed potential. For instance, Disc1 knockdown in adult dentate gyrus (DG) neurons led to increased mTOR signaling, hyperexcitability, and structural neuronal defects. These alterations were associated with significant cognitive and emotional deficits, reversible upon deactivation of affected neurons. Crucially, reversing increased mTOR signaling with an FDA‐approved inhibitor could prevent and mitigate these behavioral deficits [92]. Sodium/calcium exchange inhibitors, which regulate intracellular calcium levels—a pivotal factor in neurotransmitter release and signaling—demonstrate potential for SCZ treatment [93, 94, 95]. By modulating neuronal excitability and preventing excessive excitation, these inhibitors help maintain calcium homeostasis and could ameliorate neurotransmission abnormalities in SCZ [96, 97].

### 3.8 Protein Association Analysis

**FIGURE 7:**
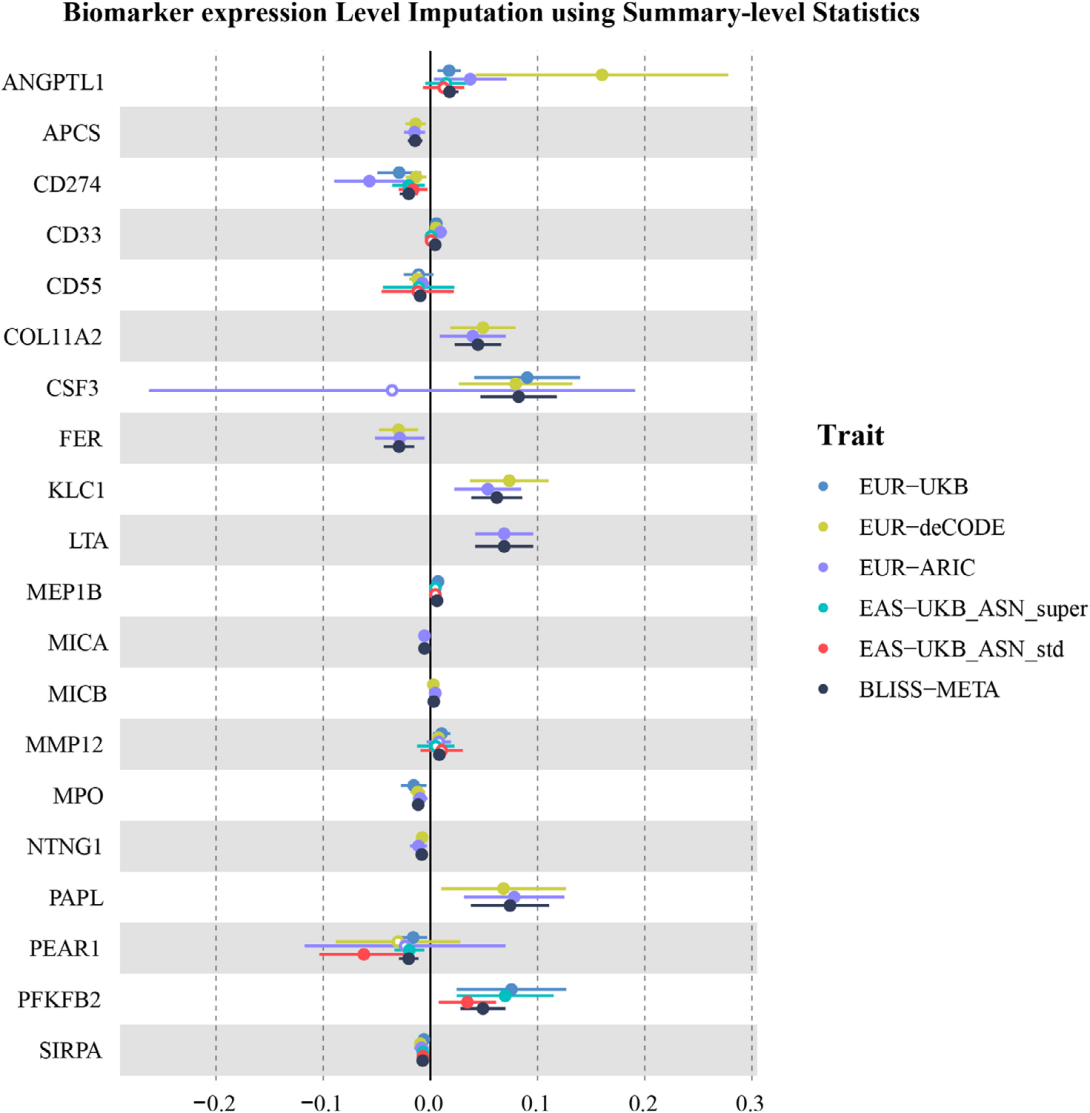
Correlations of risk protein in each pQTL dataset derived through BLISS analysis, based on Beta and SE values.

Utilizing the BLISS technique, we conducted a comprehensive analysis of plasma proteins across multiple ethnicities combined with MT EUR and MT EAS data, identifying 888 proteins with significant associations (*p* < 0.05). Our meta‐analysis highlighted 345 proteins of relevance; however, heterogeneity testing indicated that 46 proteins exhibited an *I* ^2^ greater than 40, necessitating the use of a random‐effects model, whereas the remaining proteins were analyzed using a fixed‐effects model (Table S17). Following Bonferroni correction, we identified 20 proteins significantly associated with SCZ, seven of which consistently showed statistical significance across all five included pQTL datasets (Table S18). Notably, SIRPA exhibited the most significant negative correlation with SC (*Z* = −6.41, adj. *p* = 5.02 × 10^−8^), while MICB displayed the strongest positive correlation (*Z* = 6.20, adj. *p* = 1.98 × 10^−7^) (Figure 7).

### 3.9 Multitrait PRS Modeling

All three mtPGS models showed numerically higher discriminative performance than the single‐trait SCZ model constructed using DBSLMM‐Auto (AUC = 0.562, 95% CI: 0.511–0.613). The SCZ–COPD model achieved the highest performance (AUC = 0.582, 95% CI: 0.532–0.632), followed by the SCZ–asthma (AUC = 0.579, 95% CI: 0.529–0.629) and SCZ–pneumonia models (AUC = 0.578, 95% CI: 0.527–0.628) (Table S19). We therefore further examined whether smoking status modified the effect of the best‐performing SCZ–COPD mtPGS model. The interaction between the SCZ–COPD mtPGS and smoking status was not statistically significant (*p* = 0.239), providing no evidence that its association with SCZ risk was modified by smoking.

## 4 Discussion

This study has advanced our understanding of the genetic connections between SCZ and chronic lung diseases by performing an extensive GWAS meta‐analysis across European and East Asian populations. While prior research has indicated a bidirectional association between these conditions, our work has further elucidated the genetic interplay, leveraging the largest compilations of SCZ GWAS and chronic lung disease data to date.

Our analytical approach was multifaceted and innovative. The use of multitrait analysis through MTAG not only bolstered our capability to detect genetic associations for specific disease traits but also enhanced the statistical power by exploring genetic correlations across different traits. Further, with advanced fine mapping tools such as MESuSiE, we pinpointed independent genetic variants and risk loci pertinent to SCZ, including notable discoveries like the SC‐associated SNV rs1894401.

From a functional genomics standpoint, our study didn't just identify disease‐associated genetic markers; it integrated various gene mapping techniques to highlight genes implicated in metabolic, nervous, cardiovascular, and immune system functions—all relevant to SCZ's complex etiology. Particularly notable among the 38 plausible genes were IFRD2, PCCB, and TRIM8, which showed strong associations across all analytical methods used. Intriguingly, PCCB has been linked to altered neuronal functions in human forebrain‐like organoids postknockdown, impacting pathways like γ‐aminobutyric acid (GABA)‐ergic synapses that are crucial to SCZ risk [98]. Additionally, POU3F2 may influence TRIM8 expression through the SCZ‐associated SNP rs5011218, playing a role in neurodevelopmental and synaptic functions [99].

In addition to these plausible genes, TWAS and machine learning models highlighted WBP1L and CNNM2 as important genes in SCZ pathology. These findings are consistent with previous evidence implicating WBP1L as a miR‐137 target and SCZ candidate gene, with WBP1L variants associated with SCZ risk and symptom severity [100, 101]. Compared with these earlier variant‐level association studies, our results further suggest that genetically regulated WBP1L expression may contribute to SCZ susceptibility. CNNM2 has also been repeatedly implicated in SCZ genetic studies, and previous work linked CNNM2 risk variants to social cognition‐related brain structure, ATP5MD regulation, ATP production, neurodevelopment, and magnesium homeostasis [102, 103, 104]. Our CNNM2 signal in frontal cortex BA9 therefore supports a potential role of cortical transcriptional dysregulation in SCZ. Although direct evidence connecting WBP1L and CNNM2 to chronic pulmonary diseases remains limited, their prioritization in our cross‐trait framework suggests that they may contribute to biological pathways shared between SCZ and pulmonary comorbidity.

Our machine learning analysis further supported the diagnostic relevance of these prioritized genes and underscored their potential value for understanding SCZ pathogenesis. In the realm of drug repurposing, our computational efforts suggested new therapeutic avenues, notably through mTOR inhibitors and sodium‐calcium exchange inhibitors, which hold promise for SC treatment.

The PRS analysis provided complementary evidence at the predictive level. Incorporating COPD GWAS information into the SCZ score produced the highest numerical discrimination for SCZ case‐control status (AUC = 0.582, 95% CI: 0.532–0.632), compared with the single‐trait SCZ score (AUC = 0.562, 95% CI: 0.511–0.613). The SCZ–asthma and SCZ–pneumonia models also showed slightly higher AUCs than the single‐trait model. These results indicate that pulmonary‐trait information may provide a modest incremental contribution to SCZ risk prediction. However, the confidence intervals overlapped, and the interaction between the SCZ–COPD score and smoking was not significant. The PRS findings should therefore be regarded as preliminary evidence of shared predictive information rather than clinically meaningful improvement or proof of a causal relationship.

Several limitations should be acknowledged. First, the large number of variants, genes, proteins, tissues, and cross‐trait associations evaluated may increase the risk of false‐positive findings despite the application of multiple‐testing correction and cross‐method prioritization; therefore, the identified loci, genes, and proteins require replication and functional validation. Second, phenotypes derived from registry‐ or biobank‐based diagnostic codes may be affected by incomplete ascertainment or misclassification, particularly for heterogeneous conditions such as SCZ and chronic pulmonary diseases. Third, selection bias and limited population representativeness may affect the generalizability of our findings. FinnGen reflects the genetic and healthcare characteristics of the Finnish population, whereas UK Biobank participants are generally healthier and less socioeconomically deprived than the broader population. The PRS analyses were also primarily restricted to individuals of European ancestry and showed only modest discrimination, limiting their immediate clinical applicability and transferability to other ancestries. In addition, heterogeneity across publicly available GWAS, transcriptomic, and proteomic datasets may introduce residual confounding, while summary‐level analyses cannot fully account for individual‐level environmental exposures or establish causality. Finally, the machine learning models were developed using relatively small and heterogeneous transcriptomic datasets and require validation in larger, independent clinical cohorts.

## 5 Conclusions

The genetic overlap between SCZ and chronic pulmonary diseases uncovered in this study offers insights for cross‐disorder drug repurpose and improved diagnostic models. Advanced techniques like mtPGS and multiancestry data provide a deeper understanding of SCZ's etiology and its connection to other complex traits, emphasizing the potential of genetic research to improve patient outcomes.

## Funding

The authors have nothing to report.

## Ethics Statement

The authors have nothing to report.

## Consent

All authors have consented to the publication of this manuscript.

## Conflicts of Interest

The authors declare no conflicts of interest.

## Supporting information


**Figure S1:** Left is the QQ‐plot. The *x*‐axis of the QQ‐plot represents the expected *p*‐values under the null hypothesis, while the *y*‐axis represents the observed *p*‐values from the GWAS summary statistics data. There is a significant deviation from the diagonal line, which indicates potential variations from the null hypothesis that may result from true associations or LD. The genomic inflation factor (lambda) is labeled in the top left of the QQ‐plot, and a lambda value of 1.048 indicating no strong population stratification. Right is the PZ plot, the observed *p*‐values on the *y*‐axis and the corresponding *p*‐values derived from *z*‐scores that estimated by beta and se on the *x*‐axis. A strong concordance can be observed between the observed *p*‐values and those calculated from *Z*‐scores.
**Figure S2:** Merging and de‐batching effects of five GEO data sets on schizophrenia. A and C represent before batch effect is removed, and B and D represent after batch effect is removed.
**Figure S3:** The highest rated gene among the nine machine learning models.

**Table S1:** Summary of the GWAS datasets, genome builds, ancestry groups, and sample sizes used for schizophrenia and pulmonary phenotypes.
**Table S2:** Characteristics of the GEO transcriptomic datasets used for expression validation and machine learning analyses.
**Table S3:** Quality‐control and meta‐analysis statistics for the included GWAS datasets, including sample‐overlap and genomic‐inflation metrics.
**Table S4:** Genome‐wide genetic correlations between schizophrenia and pulmonary phenotypes estimated using LDSC in Europeans and GNOVA in East Asians.
**Table S5:** Genetic correlations between schizophrenia and pulmonary phenotypes after conditioning the pulmonary GWAS associations on cigarettes smoked per day.
**Table S6:** Independent genome‐wide significant variants and their functional annotations from the multiancestry and multitrait analysis.
**Table S7:** Smoking‐related pleiotropic variants identified by mtCOJO for the pulmonary phenotypes included in the conditional analyses.
**Table S8:** Bayesian fine‐mapping results and posterior inclusion probabilities for the independent variants identified by the multitrait analysis.
**Table S9:** Ancestry‐specific and shared posterior inclusion probabilities estimated by MESuSiE across European and East Asian populations.
**Table S10:** Genes mapped to the multiancestry and multitrait association signals using the FUMA annotation framework.
**Table S11:** Gene‐level association results for schizophrenia obtained using MAGMA.
**Table S12:** Gene‐level association results for schizophrenia obtained using the mBAT‐combo method.
**Table S13:** Transcriptome‐wide association results across the evaluated tissues for the cross‐trait‐enhanced schizophrenia GWAS.
**Table S14:** Summary‐data‐based Mendelian randomization results identifying genes with pleiotropic associations with schizophrenia across tissues.
**Table S15:** Mammalian phenotype ontology enrichment results for the high‐confidence schizophrenia genes.
**Table S16:** Connectivity map compounds with transcriptional signatures inversely connected to the schizophrenia‐associated gene‐expression profile.
**Table S17:** Meta‐analysis results for proteins associated with schizophrenia across the included plasma pQTL datasets.
**Table S18:** Proteins significantly associated with schizophrenia after Bonferroni correction across the plasma pQTL meta‐analysis.
**Table S19:** Discriminative performance of the single‐trait and multitrait schizophrenia polygenic score models.

**Data S1:** gbb70062‐sup‐0003‐Supinfo.docx.

## Data Availability

All GWAS summary statistics and functional genomic datasets used in this study were obtained from publicly available resources. The PRS analyses were conducted through the web‐based PGSFusion platform; therefore, the authors did not apply for, download, or directly access individual‐level UK Biobank data. The data generated by the present analysis are provided within the article and its Supporting Information. Additional derived summary results are available from the corresponding author upon reasonable request.

## References

[1] A. Jablensky , “The Diagnostic Concept of Schizophrenia: Its History, Evolution, and Future Prospects,” Dialogues in Clinical Neuroscience 12 (2010): 271–287, 10.31887/DCNS.2010.12.3/ajablensky. 20954425 PMC3181977

[2] M. J. Owen , A. Sawa , and P. B. Mortensen , “Schizophrenia,” Lancet 388 (2016): 86–97, 10.1016/s0140-6736(15)01121-6. 26777917 PMC4940219

[3] H. Häfner and W. an der Heiden , “Epidemiology of Schizophrenia,” Canadian Journal of Psychiatry 42 (1997): 139–151, 10.1177/070674379704200204. 9067063

[4] R. Lozano , M. Naghavi , K. Foreman , et al., “Global and Regional Mortality From 235 Causes of Death for 20 Age Groups in 1990 and 2010: A Systematic Analysis for the Global Burden of Disease Study 2010,” Lancet 380 (2012): 2095–2128, 10.1016/s0140-6736(12)61728-0. 23245604 PMC10790329

[5] E. Chesney , G. M. Goodwin , and S. Fazel , “Risks of All‐Cause and Suicide Mortality in Mental Disorders: A Meta‐Review,” World Psychiatry 13 (2014): 153–160, 10.1002/wps.20128. 24890068 PMC4102288

[6] S. A. Stilo and R. M. Murray , “Non‐Genetic Factors in Schizophrenia,” Current Psychiatry Reports 21 (2019): 100, 10.1007/s11920-019-1091-3. 31522306 PMC6745031

[7] S. Jauhar , M. Johnstone , and P. J. McKenna , “Schizophrenia,” Lancet 399 (2022): 473–486, 10.1016/s0140-6736(21)01730-x. 35093231

[8] G. B. Chand , P. Singhal , D. B. Dwyer , et al., “Schizophrenia Imaging Signatures and Their Associations With Cognition, Psychopathology, and Genetics in the General Population,” American Journal of Psychiatry 179 (2022): 650–660, 10.1176/appi.ajp.21070686. 35410495 PMC9444886

[9] K. Partti , T. Vasankari , M. Kanervisto , et al., “Lung Function and Respiratory Diseases in People With Psychosis: Population‐Based Study,” British Journal of Psychiatry 207 (2015): 37–45, 10.1192/bjp.bp.113.141937. 25858177

[10] S. Suetani , F. Honarparvar , D. Siskind , et al., “Increased Rates of Respiratory Disease in Schizophrenia: A Systematic Review and Meta‐Analysis Including 619,214 Individuals With Schizophrenia and 52,159,551 Controls,” Schizophrenia Research 237 (2021): 131–140, 10.1016/j.schres.2021.08.022. 34521040

[11] N. Launders , L. Kirsh , D. P. J. Osborn , and J. F. Hayes , “The Temporal Relationship Between Severe Mental Illness Diagnosis and Chronic Physical Comorbidity: A UK Primary Care Cohort Study of Disease Burden Over 10 Years,” Lancet Psychiatry 9 (2022): 725–735, 10.1016/s2215-0366(22)00225-5. 35871794 PMC9630158

[12] X. Y. Zhang , Y. L. Tan , D. F. Zhou , et al., “Nicotine Dependence, Symptoms and Oxidative Stress in Male Patients With Schizophrenia,” Neuropsychopharmacology 32 (2007): 2020–2024, 10.1038/sj.npp.1301317. 17228336

[13] I. Panaccione , F. Napoletano , A. Forte , et al., “Neurodevelopment in Schizophrenia: The Role of the Wnt Pathways,” Current Neuropharmacology 11 (2013): 535–558, 10.2174/1570159x113119990037. 24403877 PMC3763761

[14] S. Reuter , H. Beckert , and C. Taube , “Take the Wnt Out of the Inflammatory Sails: Modulatory Effects of Wnt in Airway Diseases,” Laboratory Investigation 96 (2016): 177–185, 10.1038/labinvest.2015.143. 26595171

[15] C. E. Pelgrim , J. D. Peterson , H. R. Gosker , et al., “Psychological Co‐Morbidities in COPD: Targeting Systemic Inflammation, A Benefit for Both?,” European Journal of Pharmacology 842 (2019): 99–110, 10.1016/j.ejphar.2018.10.001. 30336140

[16] L. S. Sæther , T. Ueland , B. Haatveit , et al., “Inflammation and Cognition in Severe Mental Illness: Patterns of Covariation and Subgroups,” Molecular Psychiatry 28 (2023): 1284–1292, 10.1038/s41380-022-01924-w. 36577840 PMC10005942

[17] P. Sekula , M. F. Del Greco , C. Pattaro , and A. Köttgen , “Mendelian Randomization as an Approach to Assess Causality Using Observational Data,” Journal of the American Society of Nephrology 27 (2016): 3253–3265, 10.1681/asn.2016010098. 27486138 PMC5084898

[18] L. E. Duncan , H. Shen , J. S. Ballon , K. V. Hardy , D. L. Noordsy , and D. F. Levinson , “Genetic Correlation Profile of Schizophrenia Mirrors Epidemiological Results and Suggests Link Between Polygenic and Rare Variant (22q11.2) Cases of Schizophrenia,” Schizophrenia Bulletin 44 (2018): 1350–1361, 10.1093/schbul/sbx174. 29294133 PMC6192473

[19] J. G. Pouget , Schizophrenia Working Group of the Psychiatric Genomics Consortium , B. Han , et al., “Cross‐Disorder Analysis of Schizophrenia and 19 Immune‐Mediated Diseases Identifies Shared Genetic Risk,” Human Molecular Genetics 28 (2019): 3498–3513, 10.1093/hmg/ddz145. 31211845 PMC6891073

[20] R. Zhang , A. Sjölander , A. Ploner , D. Lu , C. M. Bulik , and S. E. Bergen , “Novel Disease Associations With Schizophrenia Genetic Risk Revealed in ~400,000 UK Biobank Participants,” Molecular Psychiatry 27 (2022): 1448–1454, 10.1038/s41380-021-01387-5. 34799693 PMC9106855

[21] W. Gong , P. Guo , Y. Li , et al., “Role of the Gut–Brain Axis in the Shared Genetic Etiology Between Gastrointestinal Tract Diseases and Psychiatric Disorders: A Genome‐Wide Pleiotropic Analysis,” JAMA Psychiatry 80 (2023): 360–370, 10.1001/jamapsychiatry.2022.4974. 36753304 PMC9909581

[22] N. Solovieff , C. Cotsapas , P. H. Lee , S. M. Purcell , and J. W. Smoller , “Pleiotropy in Complex Traits: Challenges and Strategies,” Nature Reviews. Genetics 14 (2013): 483–495, 10.1038/nrg3461. PMC4104202 23752797

[23] M. Verbanck , C. Y. Chen , B. Neale , and R. Do , “Detection of Widespread Horizontal Pleiotropy in Causal Relationships Inferred From Mendelian Randomization Between Complex Traits and Diseases,” Nature Genetics 50 (2018): 693–698, 10.1038/s41588-018-0099-7. 29686387 PMC6083837

[24] D. Chung , C. Yang , C. Li , J. Gelernter , and H. Zhao , “GPA: A Statistical Approach to Prioritizing GWAS Results by Integrating Pleiotropy and Annotation,” PLoS Genetics 10 (2014): e1004787, 10.1371/journal.pgen.1004787. 25393678 PMC4230845

[25] C. H. Lee , H. Shi , B. Pasaniuc , E. Eskin , and B. Han , “PLEIO: A Method to Map and Interpret Pleiotropic Loci With GWAS Summary Statistics,” American Journal of Human Genetics 108 (2021): 36–48, 10.1016/j.ajhg.2020.11.017. 33352115 PMC7820744

[26] V. Trubetskoy , A. F. Pardiñas , T. Qi , et al., “Mapping Genomic Loci Implicates Genes and Synaptic Biology in Schizophrenia,” Nature 604 (2022): 502–508, 10.1038/s41586-022-04434-5. 35396580 PMC9392466

[27] M. I. Kurki , J. Karjalainen , P. Palta , et al., “FinnGen Provides Genetic Insights From a Well‐Phenotyped Isolated Population,” Nature 613 (2023): 508–518, 10.1038/s41586-022-05473-8. 36653562 PMC9849126

[28] A. Buniello , J. A. L. MacArthur , M. Cerezo , et al., “The NHGRI‐EBI GWAS Catalog of Published Genome‐Wide Association Studies, Targeted Arrays and Summary Statistics 2019,” Nucleic Acids Research 47 (2019): D1005–d1012, 10.1093/nar/gky1120. 30445434 PMC6323933

[29] E. M. Pedersen , E. Agerbo , O. Plana‐Ripoll , et al., “ADuLT: An Efficient and Robust Time‐to‐Event GWAS,” Nature Communications 14 (2023): 5553, 10.1038/s41467-023-41210-z. PMC10492844 37689771

[30] M. Lam , C. Y. Chen , Z. Li , et al., “Comparative Genetic Architectures of Schizophrenia in East Asian and European Populations,” Nature Genetics 51 (2019): 1670–1678, 10.1038/s41588-019-0512-x. 31740837 PMC6885121

[31] Y. Han , Q. Jia , P. S. Jahani , et al., “Genome‐Wide Analysis Highlights Contribution of Immune System Pathways to the Genetic Architecture of Asthma,” Nature Communications 11 (2020): 1776, 10.1038/s41467-020-15649-3. PMC7160128 32296059

[32] S. Sakaue , M. Kanai , Y. Tanigawa , et al., “A Cross‐Population Atlas of Genetic Associations for 220 Human Phenotypes,” Nature Genetics 53 (2021): 1415–1424, 10.1038/s41588-021-00931-x. 34594039 PMC12208603

[33] J. D. Backman , A. H. Li , A. Marcketta , et al., “Exome Sequencing and Analysis of 454,787 UK Biobank Participants,” Nature 599 (2021): 628–634, 10.1038/s41586-021-04103-z. 34662886 PMC8596853

[34] S. Said , R. Pazoki , V. Karhunen , et al., “Genetic Analysis of Over Half a Million People Characterises C‐Reactive Protein Loci,” Nature Communications 13 (2022): 2198, 10.1038/s41467-022-29650-5. PMC9033829 35459240

[35] F. W. Hamilton , M. Thomas , D. Arnold , et al., “Therapeutic Potential of IL6R Blockade for the Treatment of Sepsis and Sepsis‐Related Death: A Mendelian Randomisation Study,” PLoS Medicine 20 (2023): e1004174, 10.1371/journal.pmed.1004174. 36716318 PMC9925069

[36] N. Shrine , A. G. Izquierdo , J. Chen , et al., “Multi‐Ancestry Genome‐Wide Association Analyses Improve Resolution of Genes and Pathways Influencing Lung Function and Chronic Obstructive Pulmonary Disease Risk,” Nature Genetics 55 (2023): 410–422, 10.1038/s41588-023-01314-0. 36914875 PMC10011137

[37] C. Y. Chen , T. T. Chen , Y. C. A. Feng , et al., “Analysis Across Taiwan Biobank, Biobank Japan, and UK Biobank Identifies Hundreds of Novel Loci for 36 Quantitative Traits,” Cell Genomics 3 (2023): 100436, 10.1016/j.xgen.2023.100436. 38116116 PMC10726425

[38] G. Genovese , N. B. Rockweiler , B. R. Gorman , et al., “BCFtools/Liftover: An Accurate and Comprehensive Tool to Convert Genetic Variants Across Genome Assemblies,” Bioinformatics 40 (2024), 10.1093/bioinformatics/btae038. PMC10832354 38261650

[39] G. B. Chen , S. H. Lee , M. R. Robinson , et al., “Across‐Cohort QC Analyses of GWAS Summary Statistics From Complex Traits,” European Journal of Human Genetics 25 (2016): 137–146, 10.1038/ejhg.2016.106. 27552965 PMC5159754

[40] C. Khunsriraksakul , Q. Li , H. Markus , et al., “Multi‐Ancestry and Multi‐Trait Genome‐Wide Association Meta‐Analyses Inform Clinical Risk Prediction for Systemic Lupus Erythematosus,” Nature Communications 14 (2023): 668, 10.1038/s41467-023-36306-5. PMC9905560 36750564

[41] C. J. Willer , Y. Li , and G. R. Abecasis , “METAL: Fast and Efficient Meta‐Analysis of Genomewide Association Scans,” Bioinformatics 26 (2010): 2190–2191, 10.1093/bioinformatics/btq340. 20616382 PMC2922887

[42] B. Han and E. Eskin , “Random‐Effects Model Aimed at Discovering Associations in Meta‐Analysis of Genome‐Wide Association Studies,” American Journal of Human Genetics 88 (2011): 586–598, 10.1016/j.ajhg.2011.04.014. 21565292 PMC3146723

[43] Y. Shirai , Y. Nakanishi , A. Suzuki , et al., “Multi‐Trait and Cross‐Population Genome‐Wide Association Studies Across Autoimmune and Allergic Diseases Identify Shared and Distinct Genetic Component,” Annals of the Rheumatic Diseases 81 (2022): 1301–1312, 10.1136/annrheumdis-2022-222460. 35753705 PMC9380494

[44] B. K. Bulik‐Sullivan , P. R. Loh , H. K. Finucane , et al., “LD Score Regression Distinguishes Confounding From Polygenicity in Genome‐Wide Association Studies,” Nature Genetics 47 (2015): 291–295, 10.1038/ng.3211. 25642630 PMC4495769

[45] Q. Lu , B. Li , D. Ou , et al., “A Powerful Approach to Estimating Annotation‐Stratified Genetic Covariance via GWAS Summary Statistics,” American Journal of Human Genetics 101 (2017): 939–964, 10.1016/j.ajhg.2017.11.001. 29220677 PMC5812911

[46] A. Auton , G. R. Abecasis , D. M. Altshuler, (Co‐Chair) , et al., “A Global Reference for Human Genetic Variation,” Nature 526 (2015): 68–74, 10.1038/nature15393. 26432245 PMC4750478

[47] Z. Zhu , Z. Zheng , F. Zhang , et al., “Causal Associations Between Risk Factors and Common Diseases Inferred From GWAS Summary Data,” Nature Communications 9 (2018): 224, 10.1038/s41467-017-02317-2. PMC5768719 29335400

[48] M. Liu , Y. Jiang , R. Wedow , et al., “Association Studies of up to 1.2 Million Individuals Yield New Insights Into the Genetic Etiology of Tobacco and Alcohol Use,” Nature Genetics 51 (2019): 237–244, 10.1038/s41588-018-0307-5. 30643251 PMC6358542

[49] P. Turley , R. K. Walters , O. Maghzian , et al., “Multi‐Trait Analysis of Genome‐Wide Association Summary Statistics Using MTAG,” Nature Genetics 50 (2018): 229–237, 10.1038/s41588-017-0009-4. 29292387 PMC5805593

[50] P. Guo , W. Gong , Y. Li , et al., “Pinpointing Novel Risk Loci for Lewy Body Dementia and the Shared Genetic Etiology With Alzheimer's Disease and Parkinson's Disease: A Large‐Scale Multi‐Trait Association Analysis,” BMC Medicine 20 (2022): 214, 10.1186/s12916-022-02404-2. 35729600 PMC9214990

[51] K. Watanabe , E. Taskesen , A. van Bochoven , and D. Posthuma , “Functional Mapping and Annotation of Genetic Associations With FUMA,” Nature Communications 8 (2017): 1826, 10.1038/s41467-017-01261-5. PMC5705698 29184056

[52] K. Wang , M. Li , and H. Hakonarson , “ANNOVAR: Functional Annotation of Genetic Variants From High‐Throughput Sequencing Data,” Nucleic Acids Research 38 (2010): e164, 10.1093/nar/gkq603. 20601685 PMC2938201

[53] P. Rentzsch , D. Witten , G. M. Cooper , J. Shendure , and M. Kircher , “CADD: Predicting the Deleteriousness of Variants Throughout the Human Genome,” Nucleic Acids Research 47 (2019): D886–d894, 10.1093/nar/gky1016. 30371827 PMC6323892

[54] S. Dong , N. Zhao , E. Spragins , et al., “Annotating and Prioritizing Human Non‐Coding Variants With RegulomeDB v.2,” Nature Genetics 55 (2023): 724–726, 10.1038/s41588-023-01365-3. 37173523 PMC10989417

[55] P. Gormley , V. Anttila , B. S. Winsvold , et al., “Meta‐Analysis of 375,000 Individuals Identifies 38 Susceptibility Loci for Migraine,” Nature Genetics 48 (2016): 856–866, 10.1038/ng.3598. 27322543 PMC5331903

[56] X. Meng , G. Navoly , O. Giannakopoulou , et al., “Multi‐Ancestry Genome‐Wide Association Study of Major Depression Aids Locus Discovery, Fine Mapping, Gene Prioritization and Causal Inference,” Nature Genetics 56 (2024): 222–233, 10.1038/s41588-023-01596-4. 38177345 PMC10864182

[57] B. Gao and X. Zhou , “MESuSiE Enables Scalable and Powerful Multi‐Ancestry Fine‐Mapping of Causal Variants in Genome‐Wide Association Studies,” Nature Genetics 56 (2024): 170–179, 10.1038/s41588-023-01604-7. 38168930 PMC11849347

[58] A. Li , S. Liu , A. Bakshi , et al., “mBAT‐Combo: A More Powerful Test to Detect Gene‐Trait Associations From GWAS Data,” American Journal of Human Genetics 110 (2023): 30–43, 10.1016/j.ajhg.2022.12.006. 36608683 PMC9892780

[59] C. A. de Leeuw , J. M. Mooij , T. Heskes , and D. Posthuma , “MAGMA: Generalized Gene‐Set Analysis of GWAS Data,” PLoS Computational Biology 11 (2015): e1004219, 10.1371/journal.pcbi.1004219. 25885710 PMC4401657

[60] L. Dall'Aglio , C. M. Lewis , and O. Pain , “Delineating the Genetic Component of Gene Expression in Major Depression,” Biological Psychiatry 89 (2021): 627–636, 10.1016/j.biopsych.2020.09.010. 33279206 PMC7886308

[61] A. Gusev , A. Ko , H. Shi , et al., “Integrative Approaches for Large‐Scale Transcriptome‐Wide Association Studies,” Nature Genetics 48 (2016): 245–252, 10.1038/ng.3506. 26854917 PMC4767558

[62] The GTEx Consortium , “The GTEx Consortium Atlas of Genetic Regulatory Effects Across Human Tissues,” Science 369 (2020): 1318–1330, 10.1126/science.aaz1776. 32913098 PMC7737656

[63] T. Qi , Y. Wu , H. Fang , et al., “Genetic Control of RNA Splicing and Its Distinct Role in Complex Trait Variation,” Nature Genetics 54 (2022): 1355–1363, 10.1038/s41588-022-01154-4. 35982161 PMC9470536

[64] U. Võsa , A. Claringbould , H. J. Westra , et al., “Large‐Scale Cis‐ and Trans‐eQTL Analyses Identify Thousands of Genetic Loci and Polygenic Scores That Regulate Blood Gene Expression,” Nature Genetics 53 (2021): 1300–1310, 10.1038/s41588-021-00913-z. 34475573 PMC8432599

[65] J. Bryois , D. Calini , W. Macnair , et al., “Cell‐Type‐Specific Cis‐eQTLs in Eight Human Brain Cell Types Identify Novel Risk Genes for Psychiatric and Neurological Disorders,” Nature Neuroscience 25 (2022): 1104–1112, 10.1038/s41593-022-01128-z. 35915177

[66] J. Jerber , D. D. Seaton , A. S. E. Cuomo , et al., “Population‐Scale Single‐Cell RNA‐Seq Profiling Across Dopaminergic Neuron Differentiation,” Nature Genetics 53 (2021): 304–312, 10.1038/s41588-021-00801-6. 33664506 PMC7610897

[67] Z. Zhu , F. Zhang , H. Hu , et al., “Integration of Summary Data From GWAS and eQTL Studies Predicts Complex Trait Gene Targets,” Nature Genetics 48 (2016): 481–487, 10.1038/ng.3538. 27019110

[68] J. A. Blake , R. Baldarelli , J. A. Kadin , et al., “Mouse Genome Database (MGD): Knowledgebase for Mouse‐Human Comparative Biology,” Nucleic Acids Research 49 (2021): D981–d987, 10.1093/nar/gkaa1083. 33231642 PMC7779030

[69] E. Clough and T. Barrett , “The Gene Expression Omnibus Database,” Methods in Molecular Biology 1418 (2016): 93–110, 10.1007/978-1-4939-3578-9_5. 27008011 PMC4944384

[70] W. E. Johnson , C. Li , and A. Rabinovic , “Adjusting Batch Effects in Microarray Expression Data Using Empirical Bayes Methods,” Biostatistics 8 (2007): 118–127, 10.1093/biostatistics/kxj037. 16632515

[71] Z. Wang , A. Lachmann , A. B. Keenan , and A. Ma'ayan , “L1000FWD: Fireworks Visualization of Drug‐Induced Transcriptomic Signatures,” Bioinformatics 34 (2018): 2150–2152, 10.1093/bioinformatics/bty060. 29420694 PMC6454499

[72] C. Wu , Z. Zhang , X. Yang , and B. Zhao , “Large‐Scale Imputation Models for Multi‐Ancestry Proteome‐Wide Association Analysis,” bioRxiv (2023): 2023.2010.2005.561120, 10.1101/2023.10.05.561120.

[73] W. Viechtbauer , “Conducting Meta‐Analyses in R With the Metafor Package,” Journal of Statistical Software 36 (2010): 1–48, 10.18637/jss.v036.i03.

[74] S. Yang , X. Ye , X. Ji , et al., “PGSFusion Streamlines Polygenic Score Construction and Epidemiological Applications in Biobank‐Scale Cohorts,” Genome Medicine 17 (2025): 77, 10.1186/s13073-025-01505-w. 40653480 PMC12257662

[75] C. Xu , S. K. Ganesh , and X. Zhou , “mtPGS: Leverage Multiple Correlated Traits for Accurate Polygenic Score Construction,” American Journal of Human Genetics 110 (2023): 1673–1689, 10.1016/j.ajhg.2023.08.016. 37716346 PMC10577082

[76] B. Gao , C. Yang , J. Liu , and X. Zhou , “Accurate Genetic and Environmental Covariance Estimation With Composite Likelihood in Genome‐Wide Association Studies,” PLoS Genetics 17 (2021): e1009293, 10.1371/journal.pgen.1009293. 33395406 PMC7808654

[77] S. Yang and X. Zhou , “Accurate and Scalable Construction of Polygenic Scores in Large Biobank Data Sets,” American Journal of Human Genetics 106 (2020): 679–693, 10.1016/j.ajhg.2020.03.013. 32330416 PMC7212266

[78] G. B. Ehret , P. B. Munroe , K. M. Rice , et al., “Genetic Variants in Novel Pathways Influence Blood Pressure and Cardiovascular Disease Risk,” Nature 478 (2011): 103–109, 10.1038/nature10405. 21909115 PMC3340926

[79] N. Kato , M. Loh , F. Takeuchi , et al., “Trans‐Ancestry Genome‐Wide Association Study Identifies 12 Genetic Loci Influencing Blood Pressure and Implicates a Role for DNA Methylation,” Nature Genetics 47 (2015): 1282–1293, 10.1038/ng.3405. 26390057 PMC4719169

[80] C. C. Fan , R. Loughnan , C. Makowski , et al., “Multivariate Genome‐Wide Association Study on Tissue‐Sensitive Diffusion Metrics Highlights Pathways That Shape the Human Brain,” Nature Communications 13 (2022): 2423, 10.1038/s41467-022-30110-3. PMC9065144 35505052

[81] C. M. Francis , M. E. Futschik , J. Huang , et al., “Genome‐Wide Associations of Aortic Distensibility Suggest Causality for Aortic Aneurysms and Brain White Matter Hyperintensities,” Nature Communications 13 (2022): 4505, 10.1038/s41467-022-32219-x. PMC9349177 35922433

[82] Z. Sha , D. Schijven , S. E. Fisher , and C. Francks , “Genetic Architecture of the White Matter Connectome of the Human Brain,” Science Advances 9 (2023): eadd2870, 10.1126/sciadv.add2870. 36800424 PMC9937579

[83] C. Li , T. Yang , R. Ou , and H. Shang , “Overlapping Genetic Architecture Between Schizophrenia and Neurodegenerative Disorders,” Frontiers in Cell and Developmental Biology 9 (2021): 797072, 10.3389/fcell.2021.797072. 35004692 PMC8740133

[84] G. Ursini , P. di Carlo , S. Mukherjee , et al., “Prioritization of Potential Causative Genes for Schizophrenia in Placenta,” Nature Communications 14 (2023): 2613, 10.1038/s41467-023-38140-1. PMC10185564 37188697

[85] J. Yang , T. Chen , L. Sun , et al., “Potential Metabolite Markers of Schizophrenia,” Molecular Psychiatry 18 (2013): 67–78, 10.1038/mp.2011.131. 22024767 PMC3526727

[86] E. A. Ermakov , M. M. Melamud , V. N. Buneva , and S. A. Ivanova , “Immune System Abnormalities in Schizophrenia: An Integrative View and Translational Perspectives,” Frontiers in Psychiatry 13 (2022): 880568, 10.3389/fpsyt.2022.880568. 35546942 PMC9082498

[87] J. Krøll , “Schizophrenia and Liver Dysfunction,” Medical Hypotheses 56 (2001): 634–636, 10.1054/mehy.2000.1254. 11388781

[88] S. P. Liu , C. C. Lin , H. C. Lin , Y. H. Chen , and H. J. Yu , “Association Between Schizophrenia and Urinary Calculi: A Population‐Based Case‐Control Study,” PLoS One 8 (2013): e56942, 10.1371/journal.pone.0056942. 23505416 PMC3591426

[89] S. Paba , R. Frau , S. C. Godar , P. Devoto , F. Marrosu , and M. Bortolato , “Steroid 5α‐Reductase as a Novel Therapeutic Target for Schizophrenia and Other Neuropsychiatric Disorders,” Current Pharmaceutical Design 17 (2011): 151–167, 10.2174/138161211795049589. 21361868

[90] M. Kondej , P. Stępnicki , and A. A. Kaczor , “Multi‐Target Approach for Drug Discovery Against Schizophrenia,” International Journal of Molecular Sciences 19 (2018): 3105, 10.3390/ijms19103105. 30309037 PMC6213273

[91] L. Ryskalin , F. Limanaqi , A. Frati , C. L. Busceti , and F. Fornai , “mTOR‐Related Brain Dysfunctions in Neuropsychiatric Disorders,” International Journal of Molecular Sciences 19 (2018): 2226, 10.3390/ijms19082226. 30061532 PMC6121884

[92] M. Zhou , W. Li , S. Huang , et al., “mTOR Inhibition Ameliorates Cognitive and Affective Deficits Caused by Disc1 Knockdown in Adult‐Born Dentate Granule Neurons,” Neuron 77 (2013): 647–654, 10.1016/j.neuron.2012.12.033. 23439118 PMC3586374

[93] K. Opuni and J. P. Reeves , “Feedback Inhibition of Sodium/Calcium Exchange by Mitochondrial Calcium Accumulation,” Journal of Biological Chemistry 275 (2000): 21549–21554, 10.1074/jbc.M003158200. 10801871

[94] T. Iwamoto , Y. Watanabe , S. Kita , and M. P. Blaustein , “Na+/Ca2+ Exchange Inhibitors: A New Class of Calcium Regulators,” Cardiovascular & Hematological Disorders Drug Targets 7 (2007): 188–198, 10.2174/187152907781745288. 17896959

[95] G. Bastioli , S. Piccirillo , P. Castaldo , et al., “Selective Inhibition of Mitochondrial Sodium‐Calcium Exchanger Protects Striatal Neurons From α‐Synuclein Plus Rotenone Induced Toxicity,” Cell Death & Disease 10 (2019): 80, 10.1038/s41419-018-1290-6. 30692508 PMC6349907

[96] S. Sanchez‐Armass and M. P. Blaustein , “Role of Sodium‐Calcium Exchange in Regulation of Intracellular Calcium in Nerve Terminals,” American Journal of Physiology 252 (1987): C595–C603, 10.1152/ajpcell.1987.252.6.C595. 3109248

[97] T. Oda , T. Kume , Y. Izumi , K. Ishihara , H. Sugmimoto , and A. Akaike , “Na+/Ca2+ Exchanger Inhibitors Inhibit Neurite Outgrowth in PC12 Cells,” Journal of Pharmacological Sciences 116 (2011): 128–131, 10.1254/jphs.11011sc. 21521931

[98] W. Zhang , M. Zhang , Z. Xu , et al., “Human Forebrain Organoid‐Based Multi‐Omics Analyses of PCCB as a Schizophrenia Associated Gene Linked to GABAergic Pathways,” Nature Communications 14 (2023): 5176, 10.1038/s41467-023-40861-2. PMC10449845 37620341

[99] C. Ding , C. Zhang , R. Kopp , et al., “Transcription Factor POU3F2 Regulates TRIM8 Expression Contributing to Cellular Functions Implicated in Schizophrenia,” Molecular Psychiatry 26 (2021): 3444–3460, 10.1038/s41380-020-00877-2. 32929213 PMC7956165

[100] F. Guan , T. Ni , W. Han , et al., “Evaluation of the Relationships of the WBP1L Gene With Schizophrenia and the General Psychopathology Scale Based on a Case‐Control Study,” American Journal of Medical Genetics. Part B, Neuropsychiatric Genetics 183 (2020): 164–171, 10.1002/ajmg.b.32773. 31840934

[101] C. Wright , J. A. Turner , V. D. Calhoun , and N. Perrone‐Bizzozero , “Potential Impact of miR‐137 and Its Targets in Schizophrenia,” Frontiers in Genetics 4 (2013): 58, 10.3389/fgene.2013.00058. 23637704 PMC3636510

[102] Schizophrenia Working Group of the Psychiatric Genomics Consortium , “Biological Insights From 108 Schizophrenia‐Associated Genetic Loci,” Nature 511 (2014): 421–427, 10.1038/nature13595. 25056061 PMC4112379

[103] E. J. Rose , A. Hargreaves , D. Morris , et al., “Effects of a Novel Schizophrenia Risk Variant rs7914558 at CNNM2 on Brain Structure and Attributional Style,” British Journal of Psychiatry 204 (2014): 115–121, 10.1192/bjp.bp.113.131359. 24311551

[104] Z. Wang , Y. Zhu , L. Ye , et al., “Downregulation by CNNM2 of ATP5MD Expression in the 10q24.32 Schizophrenia‐Associated Locus Involved in Impaired ATP Production and Neurodevelopment,” NPJ Schizophrenia 7 (2021): 27, 10.1038/s41537-021-00159-y. 34021155 PMC8139961

