# Supplementary material for: Multiancestry and Multitrait GWAS Meta‐Analysis on Schizophrenia With a Sample of 322,321 Unveils Genetic Links to Chronic Lung Diseases
Source: Genes Brain Behav. 2026 Jul 13;25(4):e70062. doi: 10.1111/gbb.70062 (PMC13365368; doi:10.1111/gbb.70062)

**Supplementary Figure 1**. Left is the QQ-plot. The x-axis of the QQ-plot represents the expected p-values under the null hypothesis, while the y-axis represents the observed p-values from the GWAS summary statistics data. There is a significant deviation from the diagonal line, which indicates potential variations from the null hypothesis that may result from true associations or LD. The genomic inflation factor (lambda) is labeled in the top left of the QQ-plot, and a lambda value of 1.048 indicating no strong population stratification. Right is the PZ plot, the observed p-values on the y-axis and the corresponding p-values derived from z-scores that estimated by beta and se on the x-axis. A strong concordance can be observed between the observed p-values and those calculated from Z-scores.


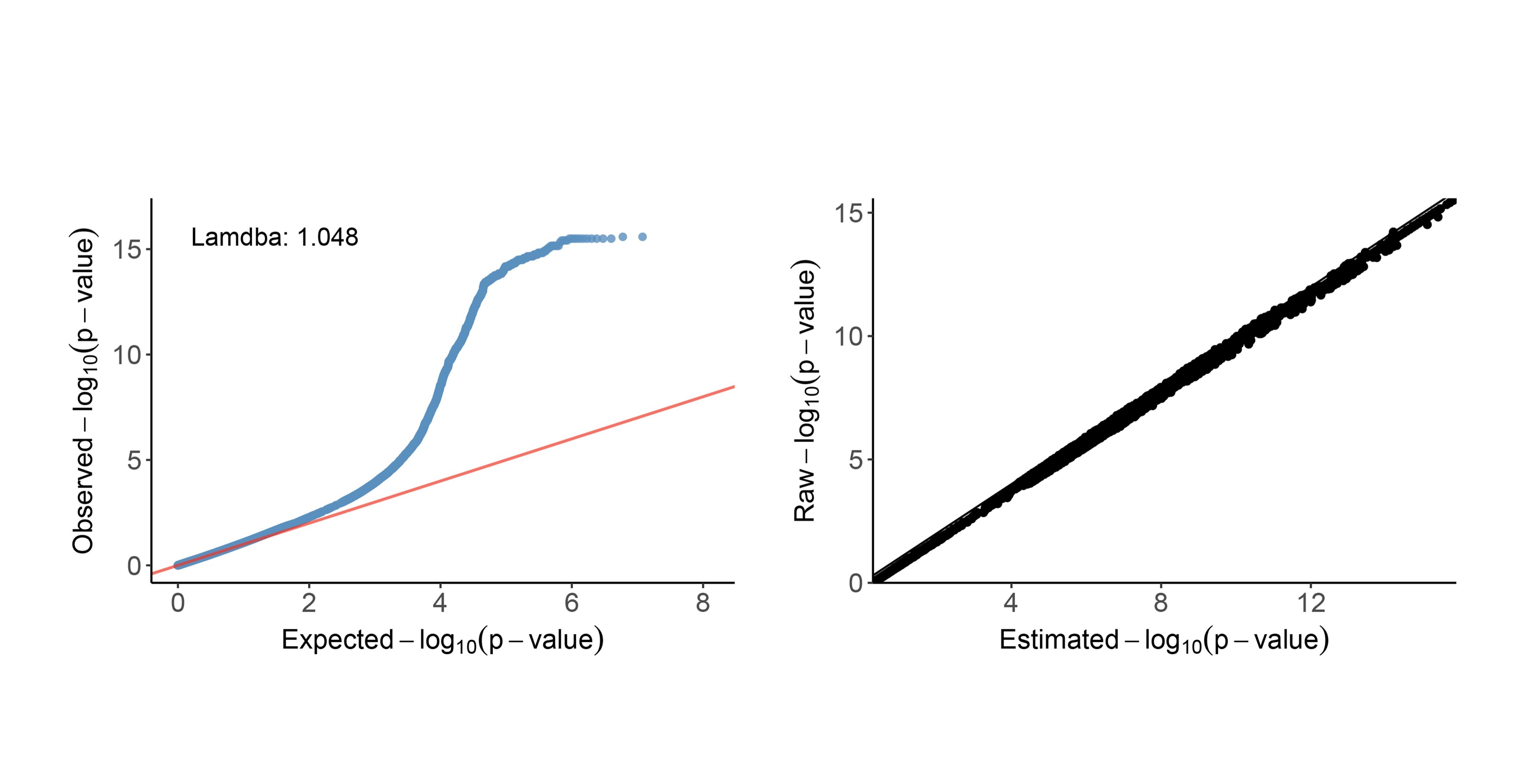


**Supplementary Figure 2.** Merging and de-batching effects of five GEO data sets on schizophrenia. A and C represent before batch effect is removed, and B and D represent after batch effect is removed.


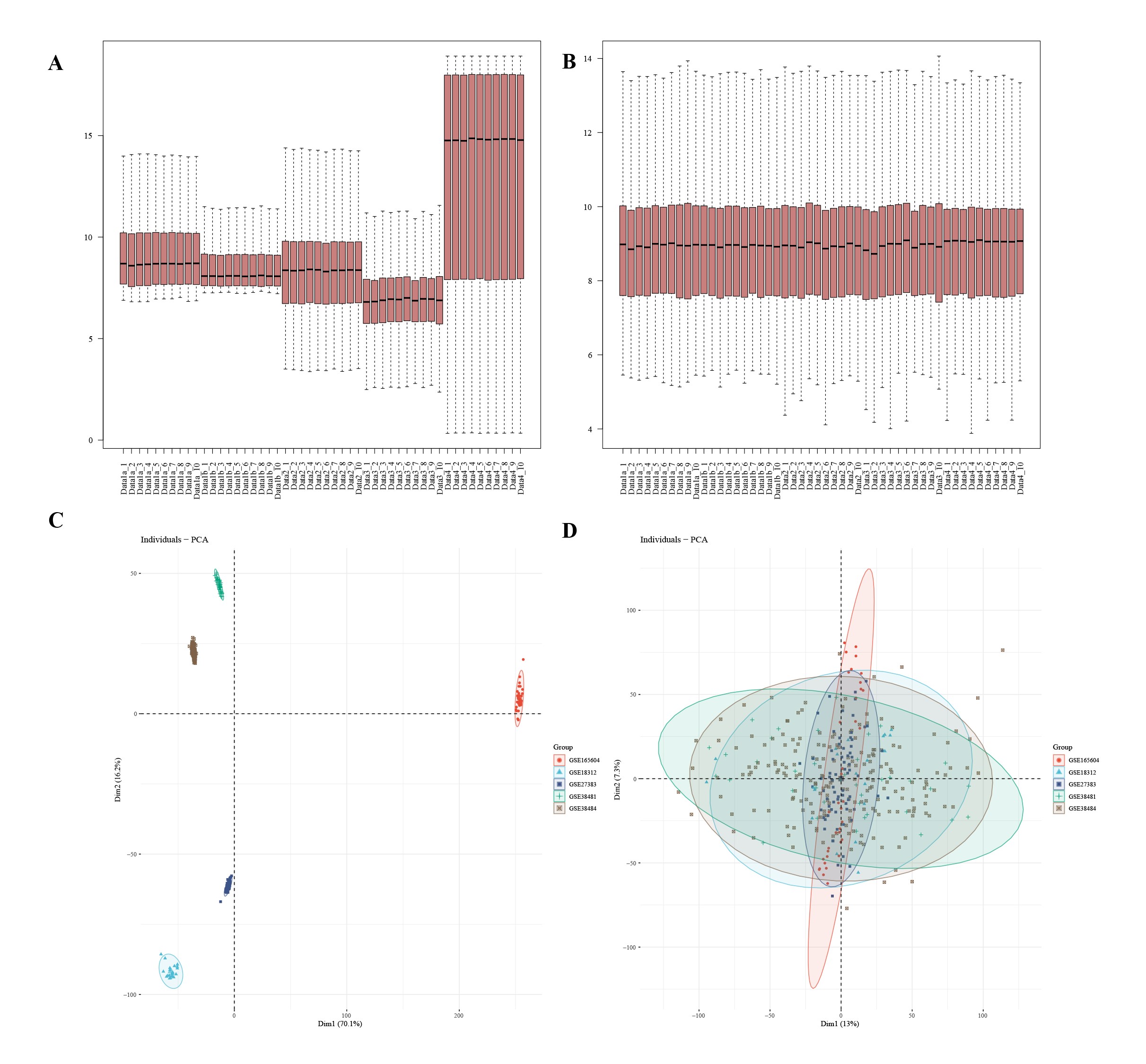


**Supplementary Figure 3.** The highest rated gene among the 9 machine learning models.


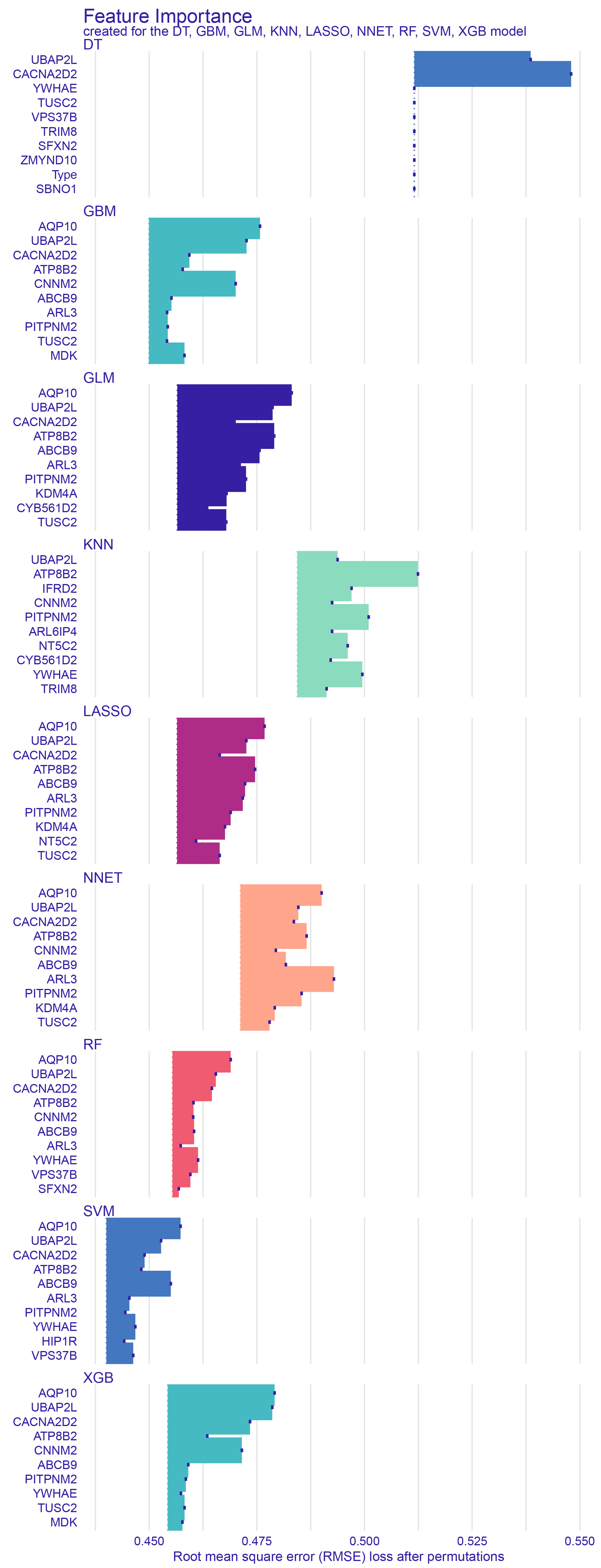

Supplement: Supplementary file 1 — Figure S1: Left is the QQ‐plot. The x‐axis of the QQ‐plot represents the expected p‐values under the null hypothesis, while the y‐axis represents the observed p‐values from the GWAS summary statistics data. There is a significant deviation from the diagonal line, which indicates potential variations from the null hypothesis that may result from true associations or LD. The genomic inflation factor (lambda) is labeled in the top left of the QQ‐plot, and a lambda value of 1.048 indicating no strong population stratification. Right is the PZ plot, the observed p‐values on the y‐axis and the corresponding p‐values derived from z‐scores that estimated by beta and se on the x‐axis. A strong concordance can be observed between the observed p‐values and those calculated from Z‐scores. Figure S2: Merging and de‐batching effects of five GEO data sets on schizophrenia. A and C represent before batch effect is removed, and B and D represent after batch effect is removed. Figure S3: The highest rated gene among the nine machine learning models. [file GBB-25-e70062-s001.docx]
