# Supplementary material for: Multiancestry and Multitrait GWAS Meta‐Analysis on Schizophrenia With a Sample of 322,321 Unveils Genetic Links to Chronic Lung Diseases
Source: Genes Brain Behav. 2026 Jul 13;25(4):e70062. doi: 10.1111/gbb.70062 (PMC13365368; doi:10.1111/gbb.70062)
Supplement: Supplementary file 3 — Data S1: gbb70062‐sup‐0003‐Supinfo.docx. [file GBB-25-e70062-s003.docx]

**G2B Author Checklist**

| The Article is clearly written in standard, scientific English language appropriate to your discipline. For help with writing in English, please visit http://wileyeditingservices.com for details of Wiley's English Language Editing Service. Please note that using this service does not guarantee acceptance of your submission by this journal. | Yes | N/A |
| --- | --- | --- |
| The Article is structured with the following required subheadings: Abstract (with no subheadings), Introduction, Material and Methods, Results, Discussion, References, Acknowledgements, Figure legends. | Yes | N/A |
| The number of subjects is clearly specified in the Methods. | Yes | N/A |
| The sex of subjects is specified. | Yes | N/A |
| For rodent studies, the genetic background is specified and the correct nomenclature ([http://www.informatics.jax.org/mgihome/nomen/](http://www.informatics.jax.org/mgihome/nomen/)) and strain abbreviations ([http://www.informatics.jax.org/mgihome/nomen/strains.shtml](http://www.informatics.jax.org/mgihome/nomen/strains.shtml)) are used. | Yes | N/A |
| For rodent studies involving mutant animals, the genetic background and breeding strategy used should follow the guidelines detailed in Crusio WE, Goldowitz D, Holmes A, Wolfer D (2009) Standards for the publication of mouse mutant studies. Genes Brain Behav 8:1-4 ([https://www.ncbi.nlm.nih.gov/pubmed/18778401](https://www.ncbi.nlm.nih.gov/pubmed/18778401)) | Yes | N/A |
| For rodent studies, housing conditions (i.e., single or group housed) are specified. | Yes | N/A |
| For rodent studies, the methods specify whether testing occurred during the light or dark phase. | Yes | N/A |
| When the same subjects received multiple tests, the order and inter-test interval is specified. | Yes | N/A |
| For human studies, the characteristics of subjects are sufficiently described (including ethnicity, age). For further guidelines on the journal’s requirements for human studies, see: [G2B guidelines](https://onlinelibrary.wiley.com/page/journal/1601183x/case-controlassociationstudies.html) | Yes | N/A |
| For human studies, there is a statement acknowledging approval by a named institutional review board and the informed consent of the subjects. | Yes | N/A |
| The Methods include a subsection describing the 'Statistical analysis'. | Yes | N/A |
| Statistical results are reported in the Results, not Figure Legends. | Yes | N/A |
| Significant p values should be accompanied by all appropriate statistical test results, including df values (example of ANOVA result: F2,20=5.14, p<.01, example of t-test result: t(9)=7.52, p<.01). | Yes | N/A |
| All figures and tables are cited in the main text of the manuscript. | Yes | N/A |
